# Extreme heat and drought at flowering could threaten global wheat yields under climate change

**DOI:** 10.1007/s10584-025-04054-8

**Published:** 2026-01-29

**Authors:** Nimai Senapati, Nigel G. Halford, Malcolm J. Hawkesford, Peter R. Shewry, Mikhail A. Semenov

**Affiliations:** Sustainable Soils and Crops, Rothamsted Research, West Common, Harpenden, AL5 2JQ UK

**Keywords:** Sirius crop model, LARS-WG stochastic weather generator, CMIP6 GCMs, Wheat reproductive development, Climate change impacts, Heat and drought stresses

## Abstract

Global food security is a major challenge in the 21st century, exacerbated by increasing frequency and intensity of extreme climatic events under climate change. The extreme climatic events around flowering, such as intense heatwaves and droughts, threaten crop yields by impacting reproductive development and grain setting. Our study estimated the potential impacts of short-term, high impact extreme temperature and drought events during flowering on global wheat yields under future climates, using climate projections from CMIP6 and a state-of-the-art wheat model (Sirius). Results show that although extreme drought around flowering currently poses a higher yield loss than extreme heat, global yield loss due to drought is predicted to decrease by 9% and 18% by 2050 and 2090, respectively. Yet, global yield losses from heat stress are expected to increase significantly, by 32% and 77% by 2050 and 2090, respectively. Consequently, heat stress at flowering emerges as a serious threat to global wheat yields under climate change, substantially increasing the vulnerability of wheat. Therefore, developing new cultivars tolerant not only to drought but also heat stress during flowering is critical for enhancing future wheat yields and sustainability in changing climates.

**Supplementary Information:**

The online version contains supplementary material available at 10.1007/s10584-025-04054-8.

## Introduction

Global food security stands out as one of the principal challenges of the 21 st century (IPCC 2019). As the global population rises towards an estimated 10 billion by 2050, the need to meet growing demand through an estimated 35% − 56% increase in food production has become an urgent concern (van Dijk et al. 2021). Rising temperature, changing precipitation patterns, and increasing frequency and magnitude of extreme events, such as extreme heat or drought, may increase the vulnerability of global food production systems (IPCC 2019; Zampieri et al. 2017). Any threat to sustainable food production by major producers and/or exporters due to climate change may result in global shortages of food, with consequent price rises, hunger, and social unrest (WFP Usa 2017).

Wheat (*Triticum aestivum* L.) is one of the world's major staple crops, providing about 20% of the global intake of calories and protein (Reynolds and Braun 2022). It plays a crucial role in global food security due to its widespread cultivation, adaptability, good storage properties and unique processing properties, and wheat-based foods are culturally embedded in wheat-producing regions. Wheat cultivation provides income and livelihoods for millions of farmers and wheat is the most widely traded of the major crops, with around 25% of global production destined for the world market (Erenstein et al. 2022).

Abiotic stresses, notably heat and water stresses, adversely impact wheat growth and development throughout the life cycle by interfering with phenological development, reducing the growing period and hence biomass production and increasing leaf senescence, reducing total photosynthesis and grain yield (Fahad et al. 2017; Asseng et al. 2015). These impacts depend on the duration and intensity of the stresses and the phenological stage of the crop at which the stresses occur. Wheat is most susceptible to abiotic stresses at the reproductive stage and short-term, low-frequency, extreme climatic events can have profound effects on reproductive development (Cseh et al. 2024; Onyemaobi et al. 2017; Barber et al. 2015; Prasad and Djanaguiraman 2014). Even short episodes of high temperature or drought around flowering carry a risk of high yield losses, with high temperature reducing yield by up to 20% for every 1 °C rise in average maximum temperature above the optimum (25–30 °C), depending on the genotype (Ullah et al. 2020; Telfer et al. 2018). Air temperatures above 30 °C for a few days during the 10-day period leading up to flowering affect meiosis, cause abnormal development of both ovary and anther, induce pollen sterility, and reduce the fertilisation rate and ultimately primary seed set number, while temperatures above 35 °C within 5–12 days after fertilization affect early endosperm development and reduce potential grain size and weight (Cseh et al. 2024; Stratonovitch and Semenov 2015; Prasad and Djanaguiraman 2014).

Drought stress also affects floret development, meiosis, gamete production, fertilization, and primary seed setting (Dong et al. 2017; Onyemaobi et al. 2017). Short episodes of severe drought within the period from 10-days before flowering until 5 days after substantially decrease the number of primary fertile seed set due to premature abortion of florets, abnormal development of reproductive organs, irreversible abortion of male and female gametes, and male and female sterility (Senapati et al. 2019; Ma et al. 2017; Onyemaobi et al. 2017; Barber et al. 2015).

The frequency and severity of short-term, extreme temperature and drought events are predicted to increase due to climate change (IPCC 2021) and understanding the impacts of such events on yields of wheat and other staple crops is therefore crucial to ensure the stability of global food production and supply. With the limited available time and resources, plant breeders may need to narrow down target cultivar traits for making genetic adaptation and yield improvement under climate change. However, the inherent unpredictability of extreme weather patterns in a changing climate makes this a major challenge. There is a large uncertainty in future climate projections between Global Climate Models (GCMs), such as in the latest Coupled Model Intercomparison Project Phase 6 (CMIP6) model ensemble (IPCC 2021; Eyring et al. 2016). Therefore, it is important for impact assessment to consider several GCMs from the CMIP6 ensemble to account for this uncertainty in climate projections. Furthermore, it is almost impossible to study the impacts of short-term and rare, extreme climatic events on reproductive development and yield in wheat experimentally at a global scale, because this would require testing of all possible combinations of future climates across different global wheat growing environments. As an alternative, well-tested, process-based crop models can be used to estimate such potential impacts globally (Asseng et al. 2015).

Many local, national, regional, and global studies have predicted the impacts of climate change on wheat growth and yields, including the impacts of heat and drought stresses (Pequeno et al. 2021; Asseng et al. 2019; Webber et al. 2018). However, global predictions of the potential impacts of low frequency, short-term, but high impact extreme climatic events, such as episodes of extreme high temperature or extreme drought, particularly around wheat flowering, are scarce (Senapati et al. 2021; Stratonovitch and Semenov 2015). The objective of the present study was therefore to estimate the potential impacts of short-term, extreme high temperatures and extreme droughts around flowering on global wheat yields in near- (2050) and far- (2090) future climates and identify priority target for wheat adaptation, using climate projections from the latest CMIP6 ensemble downscaled by the LARS-WG stochastic weather generator (Gitau et al. 2018; Semenov and Stratonovitch 2015) and the Sirius state-of-the-art wheat model (Semenov 2021; Senapati et al. 2019).

## Methods

### Global study sites

A total of 53 representative sites were selected from 33 wheat-growing countries to cover almost all the global wheat growing environments and the major producers (Supplementary Fig. S1), representing about 91% of the current global wheat-growing area and grain production (Senapati et al. 2022; FAOSTAT 2021). The site characteristics and site-specific details, including climate, management practice, and local cultivars, can be found in Supplementary Tables S1 and S2.

### Baseline climatic scenarios

The 31 years (1985–2015) of daily observed weather data were collected from local meteorological stations at each study site. To generate a baseline, observed weather data were used to estimate site-specific climatic parameters needed for a stochastic weather generator, LARS-WG 8.0 (Semenov 2024; Semenov and Stratonovitch 2015; Semenov et al. 2010). To account for yield variation due to interannual climatic variability and extreme events, 100 years of daily weather at each site were generated by using LARS-WG based on site parameters, hereafter defined as the ‘baseline climate’ (Supplementary Fig. S2). The baseline climate has statistical characteristics similar to the observed weather at each site, with probability distributions close to those of the observed climate. The accurate reproduction of climatic variability and extreme weather events by LARS-WG has been demonstrated in various studies (Gitau et al. 2018; Semenov et al. 2010). The use of 100 years of baseline climate makes the present study comparable with other studies carried out around the world, including climate change impacts (Putelat et al. 2021; Trnka et al. 2014). An atmospheric CO_2_ concentration [CO_2_] of 363.8 ppm was used for the baseline climate. The mean air temperature and cumulative precipitation over the wheat growing season under baseline climate varied from 5 to 24 °C and from 1 to 766 mm, respectively (Supplementary Table S1).

### Future climate scenarios based on the CMIP6 ensemble

Future climate projections from 15 Global Climate Models (GCMs) from the CMIP6 ensemble (Eyring et al. 2016) were used for the present study (Supplementary Table S3 and Supplementary Fig. S2). The CMIP6 ensemble has recently been used in the Sixth Assessment Report of the Intergovernmental Panel on Climate Change (IPCC 2021). Using 15 GCMs from the CMIP6 ensemble allowed estimation of uncertainty in simulated wheat yields arising from uncertainty in the GCMs climate projections. The highest greenhouse gas (GHG) emission scenario in the upper boundary of Shared Socioeconomic Pathways (SSPs) was used as an extreme or worst possible future in this study, with an additional radiative forcing of 8.5 W m^−2^ by the year 2100, viz., SSP5-8.5: Fossil-fuelled Development – Taking the Highway (high challenges to mitigation, low challenges to adaptation) (Riahi et al. 2017; O'Neill et al. 2016). Two time-frames were used as near-future (2050: period 2041–2060) and far-future (2090: period 2081–2100). The corresponding [CO_2_] of 562.8 and 1001.8 ppm were used for 2050 and 2090, respectively. LARS-WG 8.0 (Semenov 2024) was used to downscale GCM projections and generate future climate scenarios to local scale at each study site (Supplementary Fig. S2). LARS-WG downscales climate projection from GCMs and estimates delta-changes in climatic variables between future and baseline climates viz. absolute changes in monthly mean maximum and minimum temperatures, and relative changes in monthly mean precipitation and solar radiation (Semenov 2008; Semenov et al. 2025, 2024, 2010). To generate future climate scenarios, LARS-WG incorporates changes at local scale in mean climate, climatic variability and extreme events derived from the GCMs by modifying the statistical distributions of the baseline (1985–2015) weather variables at each site. The delta-changes were used to perturb site parameter distributions of LARS-WG for baseline. For each site and each GCM, 100 years of daily weather data were generated, based on GCM climate projections for the 2041 − 2060 period using LARS-WG, hereafter defined as the ‘2050-climate’ or ‘near-future climate’ (Supplementary Fig. S2). Similarly, for each site and each GCM, 100 years of daily future weather data were generated based on climate projections for the 2081–2100 period, hereafter defined as the ‘2090-climate’ or ‘far-future climate’. Adequate reproduction of climatic variability, good performances and suitability of LARS-WG weather generator in terms of capturing climate change, including extreme climatic events, at local scale, have been reported in various previous studies, including independent studies (Gitau et al. 2018; Semenov et al. 1998, 2025).

### Sirius model

Sirius 2018 (Semenov 2021) was used to simulate wheat growth, grain yield and the potential impacts of short-term, extreme droughts and extreme high temperature events around flowering under baseline and future climatic conditions. Sirius is a process-based, eco-physiological, advanced wheat model, which was calibrated for modern wheat cultivars and validated in diverse environments (Senapati et al. 2019; Stratonovitch and Semenov 2015; Lawless et al. 2005; Brooks et al. 2001; Jamieson et al. 1998). A detailed overall description of the Sirius model can be found in Supplementary methods. The impact mechanisms of extreme drought and extreme heat events around flowering on yield are described in detail as below.

#### Impact of extreme drought events around flowering

The impact mechanism of extreme drought events around flowering on primary fertile grain set number was implemented in Sirius by Senapati et al. (2019), where Sirius’s cultivar parameters related with response to short-term extreme drought stress around flowering were derived using two different studies viz. Barber et al. (2017) and Semenov et al. (2014). A short-term mechanism was implemented on an average for 15 days around flowering, viz. from 10 d before to 5 d after the flowering date, to account for the impacts of extreme drought events at flowering by using a ‘drought stress factor’ (*DSF*, dimensionless) and ‘drought reduction factor’ (*R*_*D*_, dimensionless). The *DSF* is calculated as a ratio of actual transpiration (*T*_*a*_) to potential transpiration (*T*_*p*_*)* during reproductive development.

The potential primary grain setting number per unit of ear dry mass (*N*_*pot*_, grains g^–1^) is reduced to the actual primary fertile grain setting number (*N,* grains g^–1^) due to short-term extreme drought stress around flowering as (Senapati et al. 2019):

1 $$\begin{array}{ll}N=N_{pot}\times R_D&\\R_D=1,&\mathrm{if}\;DSF>DSGNT\\R_D=DSGNRMax+S\times\left(DSF-DSGNS\right),&\mathrm{if}\;DSGNS<DSF<DSGNT\\R_D=DSGNRMax,&\mathrm{if}\;DSF\leqslant DSGNS\end{array}$$

*DSGNT* is drought stress grain number reduction threshold, while *DSGNRMax* is maximum drought stress grain number reduction, *S* is slope of the grain number reduction, in which S = (1 − *DSGNRMax*)/(*DSGNT* − *DSGNS*), and *DSGNS* is drought stress grain number reduction saturation (Supplementary Fig. S3).

#### Impact of extreme high temperature events around flowering

In Sirius, short-term extreme high temperature events around flowering affect both (i) primary fertile grain setting number and (ii) early endosperm development. The impact mechanism of extreme high temperature events around flowering on primary fertile grain set and early endosperm development was implemented in Sirius by Stratonovitch and Semenov (2015), where Sirius’s cultivar parameters related with response to short-term heat stress around flowering were calibrated and validated using experiments (Prasad and Djanaguiraman 2014) and the Hot Serial Cereal (HSC) dataset(Wall et al. 2011; White et al. 2011; Ottman et al. 2012).

(i) Primary fertile grain set

The potential primary fertile grain setting number is reduced due to short-term extreme high temperature stress around flowering by using a heat reduction factor of primary fertile grain number set, (*R*_*H*_, dimensionless). The *N*_*pot*_ is decreased to *N* due to short-term extreme high temperature around flowering as (Stratonovitch and Semenov 2015):

2 $$N={N}_{\mathrm{pot}}\times {R}_{H}$$

When the maximum canopy temperature, *T*^*A*^_max_ (^°^C), during a period from 10 days before anthesis to anthesis, which coincides with meiosis and fertilization, exceeds a threshold temperature, *T*^*N*^ (^°^C) (Stratonovitch and Semenov 2015),

3 $${R}_{H}=\mathrm{max}\left(0, \mathrm{min}\left(1, 1-\left({{T}^{A}}_{max}-{T}^{N}\right)\times {S}^{N}\right)\right)$$

where *S*^*N*^ (^◦^C^–1^) is the slope of the grain number reduction per unit of canopy temperature above *T*^*N*^ (Supplementary Fig. S4). Canopy temperature could significantly differ from air temperature depending on intercepted solar radiation, re-emitted radiation, wind speed, plant available water and rate of evapotranspiration (Javadian et al. 2024; Webber et al. 2016). Thus, using canopy temperature in model better represents the reality than air temperature in climate change impact assessments (Webber et al. 2017). In Sirius, canopy temperature is calculated from air temperature and soil surface and canopy energy balances (Webber et al. 2017; Jamieson et al. 1995).

(ii) Early endosperm development

The potential weight of a single grain (*W*_*pot*_, g grain^–1^) could be limited by heat stress during early endosperm development. The *W*_*pot*_ is reduced if the *T*^*A*^_*max*_ exceeds a threshold temperature, *T*^*W*^ (^°^C), at the beginning of grain filling; i.e., a period from 5–12 days after anthesis. The *W*_*pot*_ is reduced to actual weight of an individual grain (*W*, g grain^–1^) as:

4 $$W={W}_{pot}\times \mathrm{max}\left(0, \mathrm{min}\left(1, 1-\left({{T}^{A}}_{max}-{T}^{W}\right)\times {S}^{W}\right)\right),$$

where *S*^*W*^ (^◦^C^–1^) is the slope of the potential weight reduction per unit of canopy temperature above *T*^*W*^ (Supplementary Fig. S5).

### Model set-up

At 53 global sites, site-specific current local wheat cultivars, soils, and crop management practices, including sowing time, were used for model simulation (Supplementary Table S1 and S2, and Fig. S6). The initial soil water condition at each site was accounted in Sirius to include residual impact of precipitation in non-growing seasons or soil moisture deficit at sowing. The potential yield of current wheat cultivars (*Ycv*) in rainfed conditions under baseline climate was estimated by running the precalibrated and well-validated Sirius 2018 wheat model (Semenov 2021) (see Supplementary methods) in rainfed conditions and baseline climate under optimal crop and soil managements, *i.e*. with no yield losses due to nutrient deficiency, disease and pest infestation, or competition from weeds (Senapati et al. 2022; GYGA 2024; van Ittersum et al. 2013) (Supplementary Fig. S6). Similarly, model simulations under future climate scenarios were run with the same current local wheat cultivars, soils, and crop management practices in rainfed conditions under optimal crop and soil managements. An atmospheric [CO_2_] of 363.8 ppm was used for the baseline climate, whereas under SSP5-8.5, corresponding [CO_2_] of 562.8 and 1001.8 ppm were used in 2050 and 2090, respectively. In Sirius, *RUE* is proportional to [CO_2_], with an increase of 30% for a doubling in [CO_2_], which agrees well with different field experiments for a C3 crop such as wheat (Vanuytrecht et al. 2012). However, any potential improvement in water use efficiency resulting from elevated atmospheric CO_2_ concentration under future climate was not accounted in Sirius for the present study, due to unavailability of high-quality experimental data sets and uncertainty (Ewert et al. 2002).

### Estimation of potential impacts of short-term extreme heat and drought events around flowering

The potential impacts of short-term, low frequency extreme heat and drought around flowering both under baseline and future climate scenarios were quantified by computing 95th percentiles of two indexes, viz. 95th percentile of Heat Stress Index (HSI95p) and 95th percentile of Drought Stress Index (DSI95p) (Senapati et al. 2021, 2019; Stratonovitch and Semenov 2015; Semenov 2021) (Supplementary Fig. S6).

Heat Stress Index (HSI) is defined as (Senapati et al. 2021; Semenov 2021; Stratonovitch and Semenov 2015; Semenov and Shewry 2011):

5 $$HSI=\left(1-{Y}_{H}/Y\right),$$

where *Y* is the water-limited yield of a cultivar tolerant to short-term, extreme heat and drought stresses during reproductive development around flowering, while *Y*_H_ is the water-limited yield of a cultivar sensitive to extreme heat stress around flowering.

Drought Stress Index (DSI) is defined as (Senapati et al. 2021, 2019; Semenov 2021; Semenov and Shewry 2011):

6 $$DSI=\left(1-{Y}_{D}/Y\right),$$

where *Y*_D_ is the water-limited yield of a cultivar sensitive to extreme drought stress around flowering.

For simulation of only extreme drought impact around flowering, response mechanism of extreme high temperature was switched off in Sirius. However, any small indirect effect of change in canopy temperature on drought reduction factor *R*_*D*_, due to change in transpiration, was accounted for by drought stress factor *DSF (T*_*a*_/*T*_*p*_). On the other hand, to simulate impact of only extreme high temperature around flowering, response mechanism of extreme drought around flowering was switched off. But any indirect effect of change in canopy temperature on heat reduction factor *R*_*H*_ resulting from changes in transpiration, depending on solar radiation, air temperature and plant available water, was accounted for by simulated canopy temperature in Sirius. The cultivar tolerant to extreme heat and drought stresses during flowering was simulated in Sirius by switching off both the response mechanisms of short-term extreme drought and extreme high temperature around flowering.

HSI95p and DSI95p represent the relative yield loss due to short-term, low frequency, extreme heat stress or drought stress around flowering that could be expected to occur once every 20 years on average. Although HSI95p and DSI95p are associated with the rare yield losses events, but due to their extreme impacts on yields, global estimation of HSI95p and DSI95p beforehand under the baseline and future climatic scenarios could help in developing mitigation and adaptation strategies to ensure the stability of global food production and supply even in the rare but worst climatic years under future climate.

To estimate HSI95p and DSI95p, Sirius was run with current cultivars at each study site (Supplementary Table S1 and S2) in two different ways, viz., (a) sensitive to short-term, extreme heat and drought stresses around flowering, and (b) tolerant to short-term, extreme heat and drought around flowering (Supplementary Fig. S6). Sensitive cultivars and their yields are responsive to short-term, extreme heat (*Y*_H_) or drought stresses (*Y*_D_) around flowering, and thus affected through reduction in grain number and maximum grain size. In contrast, tolerant cultivars and their yields are insensitive to short-term, extreme heat and drought stresses around flowering (*Y*), and thus not affected by extreme heat and drought events around flowering. Both, sensitive and tolerant site-specific local cultivars were run separately under baseline and future climates in water limited conditions, with the optimal crop and soil managements as mentioned in 2.5 Model set-up. By comparing yields of the sensitive (*Y*_D_ or* Y*_H_) and tolerant (*Y*) cultivars, DSI and HSI were estimated by following the Eqs. 5 and 6. DSI95p and HSI95p were estimated by quantifying 95th percentiles of DSI and HSI, respectively (Supplementary Fig. S6).

For estimating DSI95p, the same set of cultivar parameters related to the impacts of short-term extreme drought stress around flowering on grain number and size (Supplementary Fig. S3) was used for all the cultivars at the study sites, because experimental datasets to calibrate these parameters for each individual cultivar and site were unavailable (see Supplementary methods). Similarly, for estimating HSI95p, one set of cultivar parameters related to the impacts of short-term extreme heat stress around flowering (Supplementary Figs. S4 and S5) was used for all cultivars at the study sites.

### Upscaling

Global mean potential impacts of extreme heat and drought events around flowering under future climates were estimated as a mean over study sites. Similarly, global mean wheat yield (t ha^−1^) in baseline climate was estimated as a mean over the 53 global study sites, whereas country mean was estimated as averaged over study sites available in each country. However, in order to assess the future impacts of extreme heat and drought stresses at flowering at individual country level, the most extreme site among sites available in a country was selected separately and independently each for drought and heat stress impacts to reflect the worst possible climate change impact scenario in accordance with the highest or extreme GHG emission scenario (SSP5-8.5) as used in the study.

## Results and discussion

### Potential wheat yield under baseline climate

**Fig. 1:**
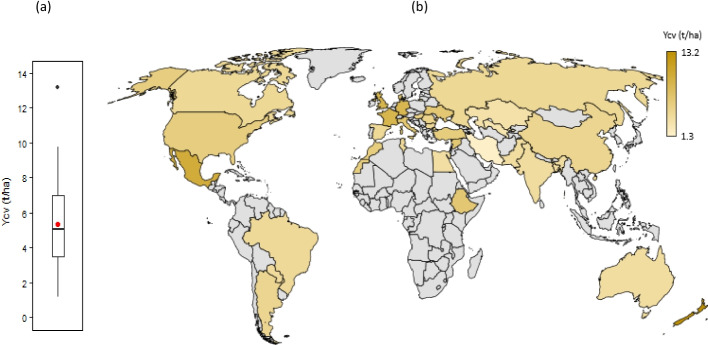
Estimated potential yield of current wheat cultivars with optimal management (*Ycv*) in rainfed conditions under baseline climate (1985–2015). (**a**) global (average over the study sites) and (**b**) in different wheat producing countries. The boxplot (a) represents the 5th percentile, 25th percentile, median, 75th percentile and 95th percentile over the 53 global study sites, with the red circle showing the mean. See Supplementary Fig. S7 for variation in potential yield within country

The mean global potential yield of current wheat cultivars with optimal management (*Ycv*) in rainfed conditions under baseline climate (1985–2015) was estimated at 5.4 t ha^−1^, with wide variation across major wheat producing countries, from 1.3 t ha^−1^ in South Africa to 13.2 t ha^−1^ in New Zealand (Fig. 1 and Supplementary Fig. S7). Among leading producers and exporters, potential wheat yields ranged from 8.6 (France), 6.5 (Ukraine), 5.8 (USA), 4.8 (China), 4.5 (Russia), 4.0 (India), 3.9 (Pakistan), to 3.8 (Australia) t ha^−1^. This variation in potential yield across different countries reflects combined influences of local environments (climate, soil etc.) and management with wheat cultivars (Supplementary Tables S1 and S2; Supplementary Fig. S8). Our estimated global averaged potential wheat yield is greater than the mean wheat yields generally achieved by farmers and reported as country yield from Food and Agriculture Organization (FAO) (FAOSTAT 2023). This is because wheat yield in our study was estimated as the potential or achievable yield of current cultivars that could be obtained if optimal crop and soil managements are achieved in rainfed conditions *i.e.,* adequate nutrient supply and effective control of diseases, pests and weeds. We estimated first potential yield of current wheat cultivars globally under a baseline climate, as our objective was to quantify potential impacts of heat and drought stress on wheat yield under future climate scenarios, for which a baseline was required. However, achieving optimal management by farmers is often challenging, due to resource and technological constrained, and diminishing returns (Schils et al. 2018; van Ittersum et al. 2013). Nevertheless, potential wheat yields as estimated in our study agree well with similar estimates (1—13 t ha^−1^) in various studies across different wheat-producing countries (Dadrasi et al. 2023a; Guarin et al. 2022; Schils et al. 2018), including most popular and comprehensive country estimates to date by the Global Yield Gap and Water Productivity Atlas (GYGA)(GYGA 2024) (Supplementary Fig. S9, correlation coefficient, *r* = 0.78^*^, and mean difference, *M* = 0.16 t ha^−1^).

### Impact of extreme drought stress around flowering on wheat yield under baseline and future climate scenarios

**Fig. 2:**
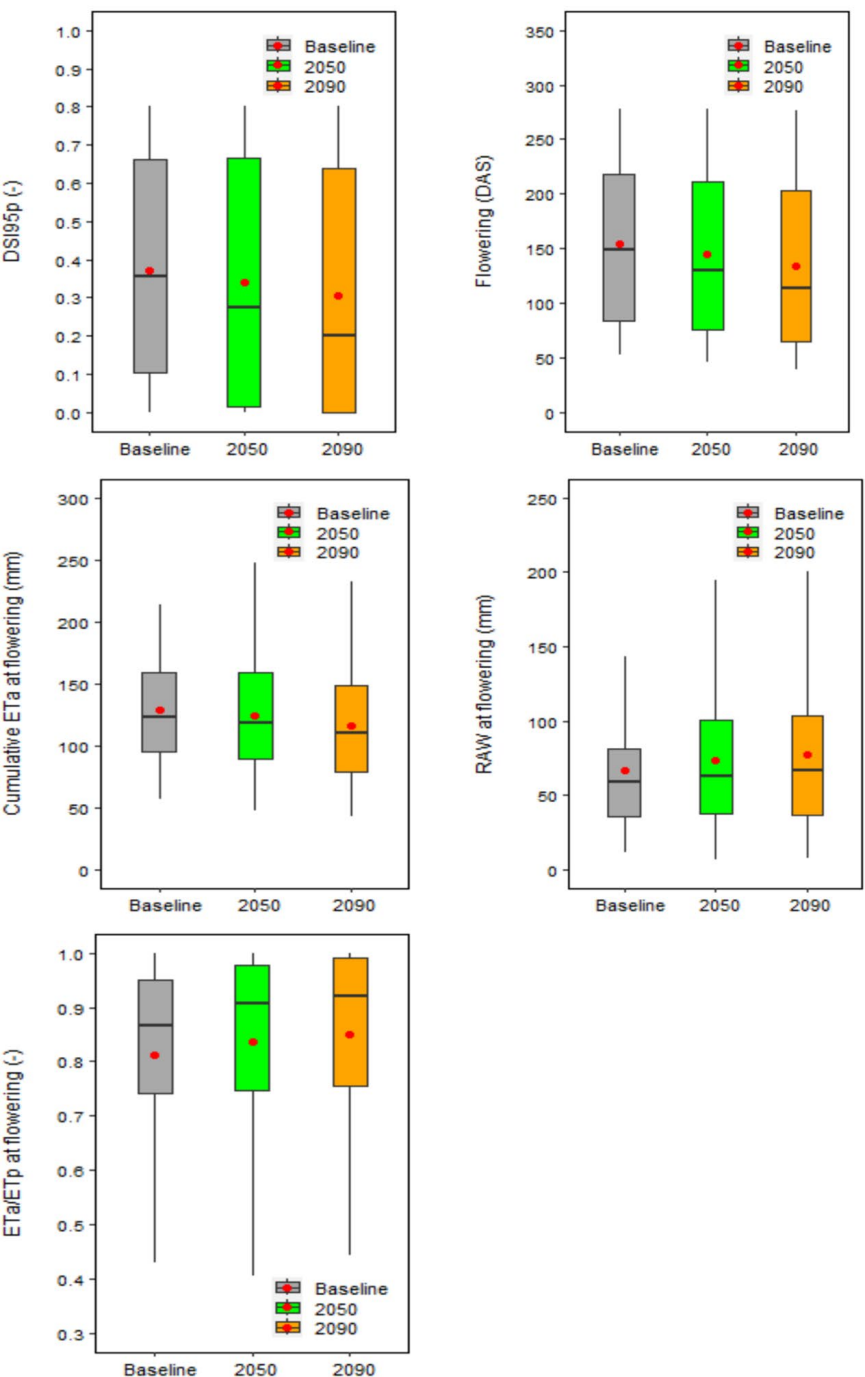
Global 95th percentile Drought Stress Index (DSI95p), mean flowering time (DAS: Days After Sowing), cumulative actual evapotranspiration at flowering (ETa, mm), root available water at flowering (RAW, mm) and ratio of cumulative actual and potential evapotranspiration at flowering (ETa/ETp) under baseline (1985–2015) and future climates (2050 and 2090). Each box plot represents the 5th percentile, 25th percentile, median, 75th percentile and 95th percentile, with the red circle showing the mean, of simulations based on 15 global climate models from the CMIP6 ensemble at 53 global study sites

**Fig. 3:**
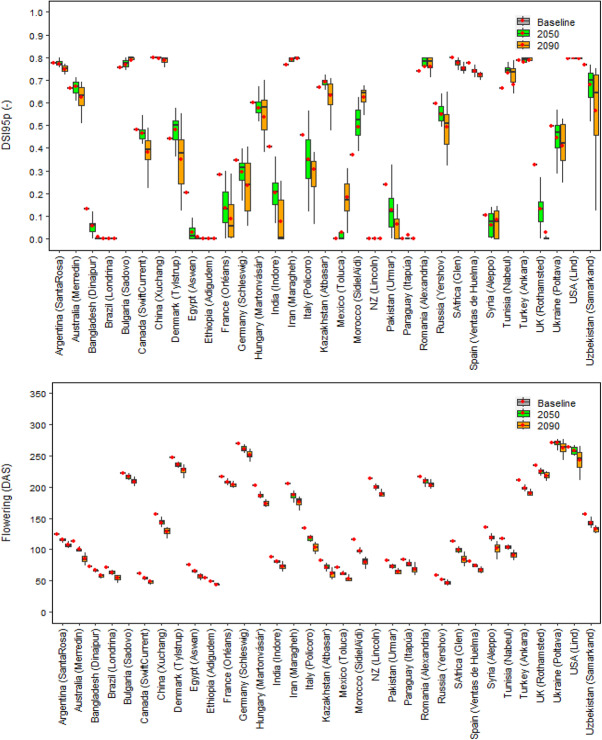

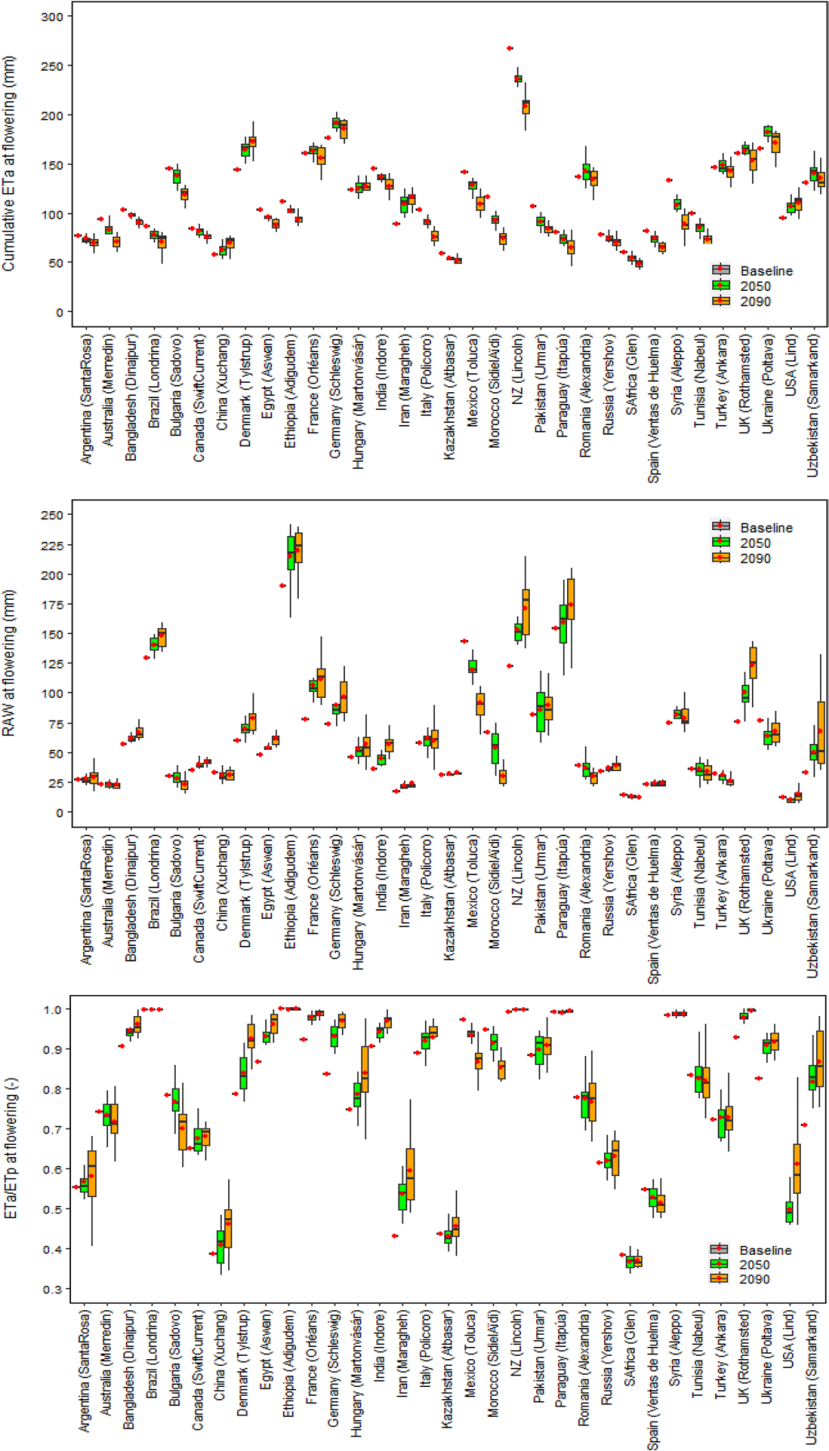
The 95th percentile Drought Stress Index (DSI95p), mean flowering time (DAS: Days After Sowing), cumulative actual evapotranspiration at flowering (ETa, mm), root available water at flowering (RAW, mm) and ratio of cumulative actual and potential evapotranspiration at flowering (ETa/ETp) in different wheat producers under baseline (1985–2015) and future climates (2050 and 2090). Each box plot represents the 5th percentile, 25th percentile, median, 75th percentile and 95th percentile, with the red circle showing the mean, of simulations based on 15 global climate models from the CMIP6 ensemble at the extreme site in a country (see Methods)

**Fig. 4:**
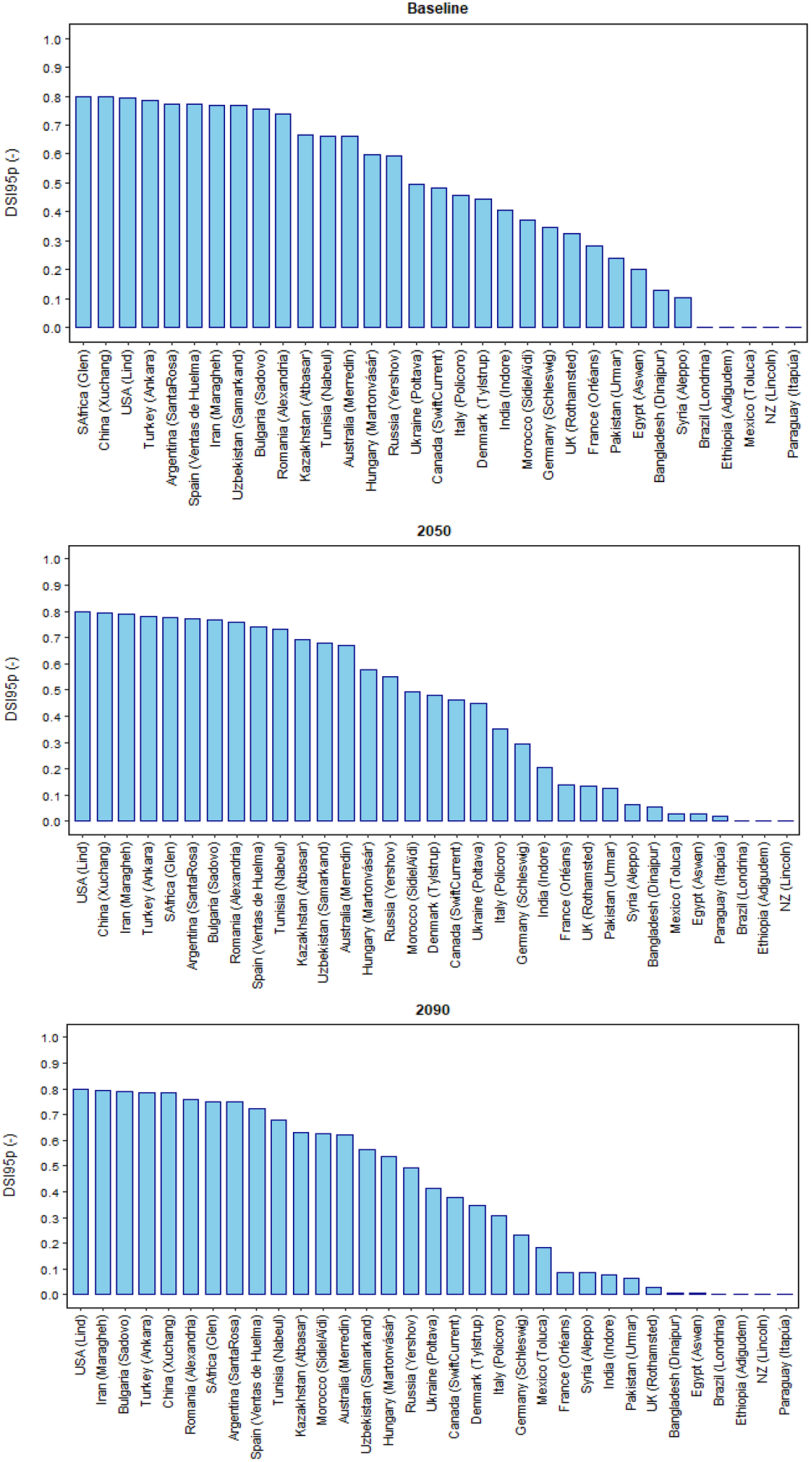
Ranking of 95^th^ percentile Drought Stress Index (DSI95p) as mean over 15 GCMs in different wheat producers under baseline (1985–2015) and future climates (2050 and 2090), based on the extreme sites (see Methods)

Under baseline climate, the mean global relative yield loss due to extreme drought events around flowering was estimated as DSI95p = 0.37 (DSI95p is the 95th percentile of the Drought Stress Index; see Methods), with a wide country variation in DSI95p of 0–0.80 (Figs. 2, 3 and 4). DSI95p represents the relative yield losses due to extreme drought stress events around flowering. The estimated DSI95p was very high (≥ 0.70) in China, South Africa, USA, Spain, Turkey, Iran, Argentina, Bulgaria, Romania and Uzbekistan. In contrast, it was small (≤ 0.10) in Brazil, Paraguay, Ethiopia, Mexico and New Zealand. A high DSI95p (0.50—0.70) was estimated in Australia, Russia and Kazakhstan, while a medium DSI95p (0.30—0.50) was estimated in Ukraine, Canada, Italy, Germany, UK and India, and a moderate DSI95p (0.10—0.30) was estimated in Egypt, Pakistan, Syria, Bangladesh and France.

Under future climatic scenarios, global DSI95p was predicted to decrease by 9% and 18% in 2050 and 2090, respectively (Fig. 2). The future warmer climate may accelerate wheat phenological development, bringing global mean wheat flowering date earlier by around 1.4 and 2.9 weeks in 2050 and 2090, respectively (Fig. 2). Hence, the average global cumulative actual evapotranspiration (*ET*_*a*_) at flowering (from sowing to flowering) was predicted to decrease by 3% and 9% in 2050 and 2090, respectively, whereas average global root available water (RAW) at flowering was predicted to increase by 10% and 16% (Fig. 2). The mean ratio of cumulative *ET*_*a*_ and potential evapotranspiration (*ET*_*p*_) at flowering was projected to increase by 3–5% under 2050 and 2090 climates (Fig. 2). Along with growing season precipitation, initial soil water condition was also accounted in Sirius to consider any residual impact of precipitation in non-growing seasons or soil water deficit at sowing (Supplementary Fig. S10). Therefore, our results indicate that despite the warmer temperature during the wheat growing season under future climates, with a minimum relative change in precipitation, earlier flowering and hence a shorter vegetative phase could result in a reduction in the cumulative evapotranspiration loss and an increase in RAW around flowering (Figs. 2 and 3, Supplementary Fig. S10). This may increase water supply/demand (ET_a_/ET_p_) ratio at flowering and reduce the impact of drought stress under future climates (Figs. 2 and 3).

To estimate the impact of drought stress at country level, where several sites were available, the most extreme site in each country was selected to gauge the worst possible climate change impacts, in accordance with the extreme GHG emission scenario (SSP5-8.5) used in this study (see Methods). The yield loss due to extreme drought around flowering was predicted to decrease in most of the wheat-producing countries under near- and far-future climates (Figs. 3 and 4); however, the absolute magnitude of the yield loss was predicted to remain high (up to DSI95p: 0.70 ~ 0.80) in a few countries; viz. Argentina, China, Bulgaria, Turkey, Iran, USA, Romania, South Africa and Spain. The profound effect of drought stress at flowering even under future climates in those countries could be explained by a low RAW (≤ 50 mm) and a lower ET_a_/ET_p_ ratio (< 0.7) at flowering (Fig. 3). Conversely, a lesser impact of drought stress under future climates in relatively favourable wheat producers, such as Brazil, Ethiopia, New Zealand and Paraguay, could be linked to a higher RAW (> 125 mm) and greater ET_a_/ET_p_ ratio (~ 1.0) at flowering (Fig. 3). The differences between countries with respect to DSI95p, RAW and ET_a_/ET_p_ ratio at flowering could be explained by variation in different combinations of soil available water capacity (SAWC), initial soil moisture deficit (SMD), growing season precipitation, air temperature, evapotranspiration and the wheat varieties grown (Figs. 3 and 4; Supplementary Fig. S10). In contrast, yield loss due to severe drought at flowering was predicted to increase in a few minor producers, notably Bulgaria, Mexico, Morocco, Iran, Romania and Tunisia (Figs. 3 and 4). This is because RAW at flowering was predicted to decrease in these countries, despite early flowering, mainly due to a substantial reduction in precipitation compared to baseline climate during the wheat season, as predicted by 15 Global Climate Models (GCMs) from the Climate Model Intercomparison Project CMIP6 ensemble (Fig. 3, and Supplementary Fig. S10).

Accelerated wheat phenology under warmer climate has been observed in different countries, including Europe (Rezaei et al. 2015). A few studies indicated that wheat water deficit could be partially avoided by accelerated phenology and precocity of phenological stages under climate change (Le Roux et al. 2024; Shavrukov et al. 2017). Early flowering has been indicated previously as a potential drought escape mechanism in water-limited environments (Hickey et al. 2022; Shavrukov et al. 2017). Thus, the impact of short-term extreme drought events around flowering on mean global wheat yield under near- and far-future climates could be partially mitigated by the reduced evapotranspiration loss, increased RAW and greater water supply/demand (ET_a_/ET_p_) ratio brought about by early flowering (Kalra et al. 2023; Hickey et al. 2022). Hence, reduced or limited evapotranspiration before flowering, improved root available water at flowering, and early flowering could be helpful traits for improving tolerance to extreme drought stresses at flowering (Kalra et al. 2023; Hickey et al. 2022; Collins et al. 2021; Senapati et al. 2020; Shavrukov et al. 2017).

### Impact of extreme heat stress around flowering on wheat yield under baseline and future climate scenarios

**Fig. 5:**
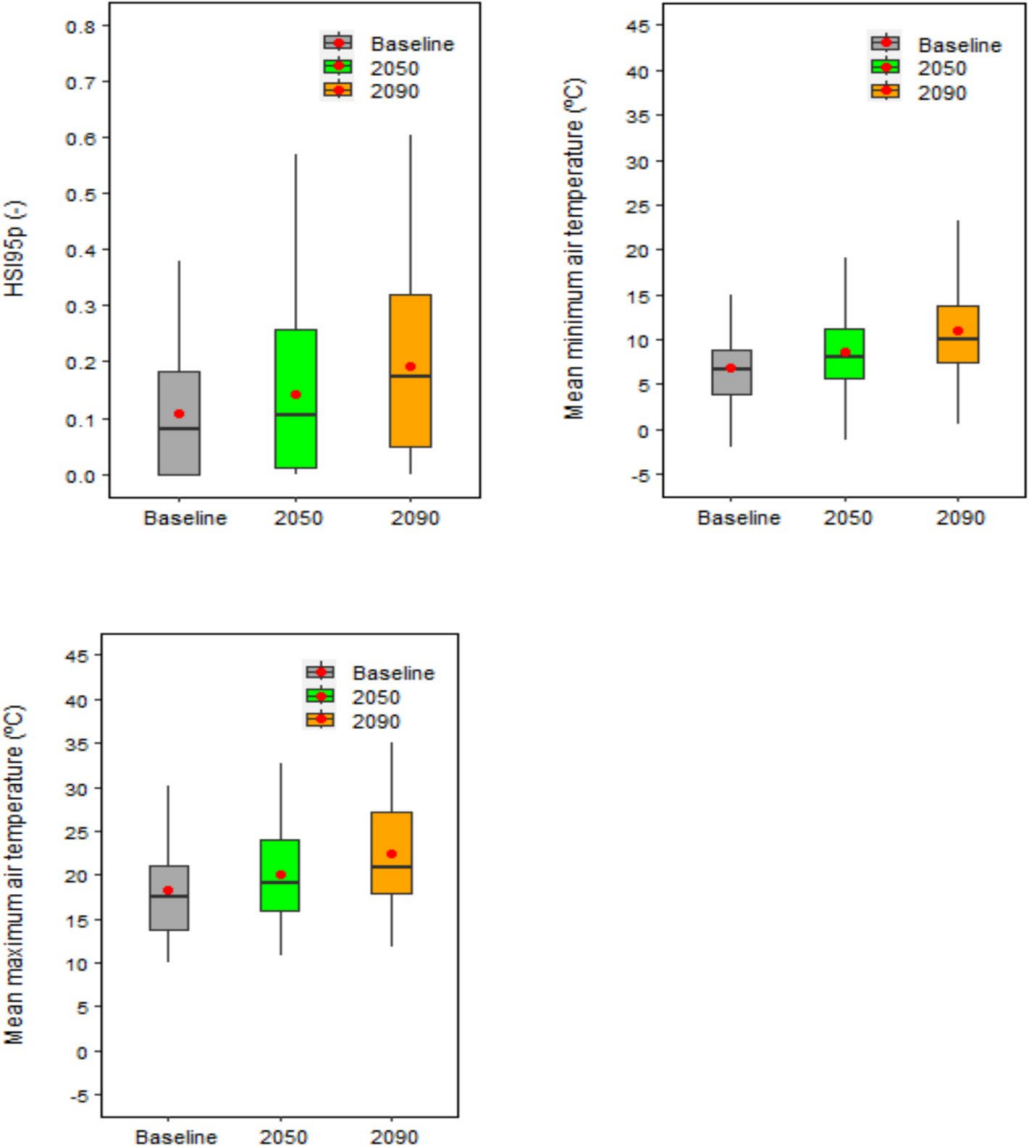
Global 95th percentile Heat Stress Index (HSI95p), and mean minimum and maximum air temperature over the wheat growing season under baseline (1985–2015) and future climates (2050 and 2090). Each box plot represents the 5th percentile, 25th percentile, median, 75th percentile and 95th percentile, with the red circle showing the mean, of simulations based on 15 global climate models from the CMIP6 ensemble at 53 global study sites

**Fig. 6:**
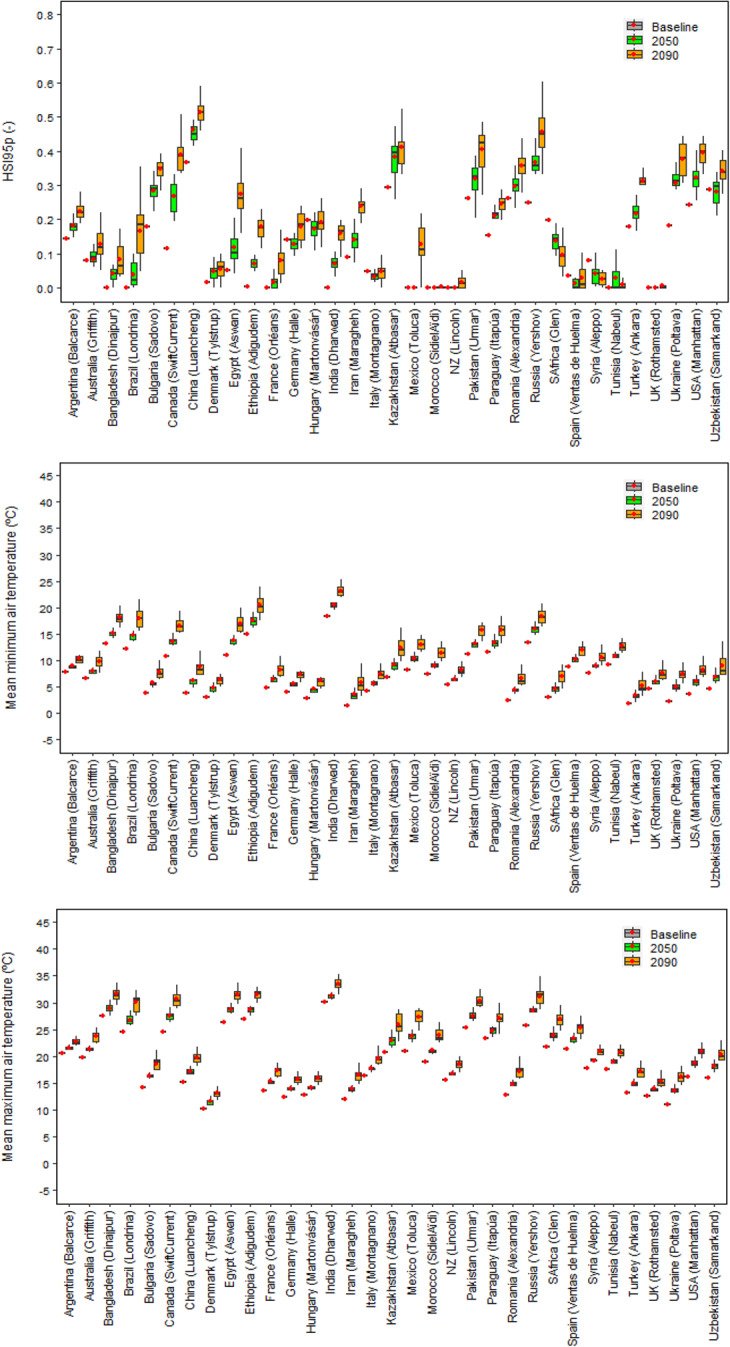
The 95th percentile Heat Stress Index (HSI95p), and mean minimum and maximum air temperature over the wheat growing season in different wheat producers under baseline (1985–2015) and future climates (2050 and 2090). Each box plot represents the 5th percentile, 25th percentile, median, 75th percentile and 95th percentile, with the red circle showing the mean, of simulations based on 15 global climate models from the CMIP6 ensemble at the extreme site in a country (see Methods)

**Fig. 7:**
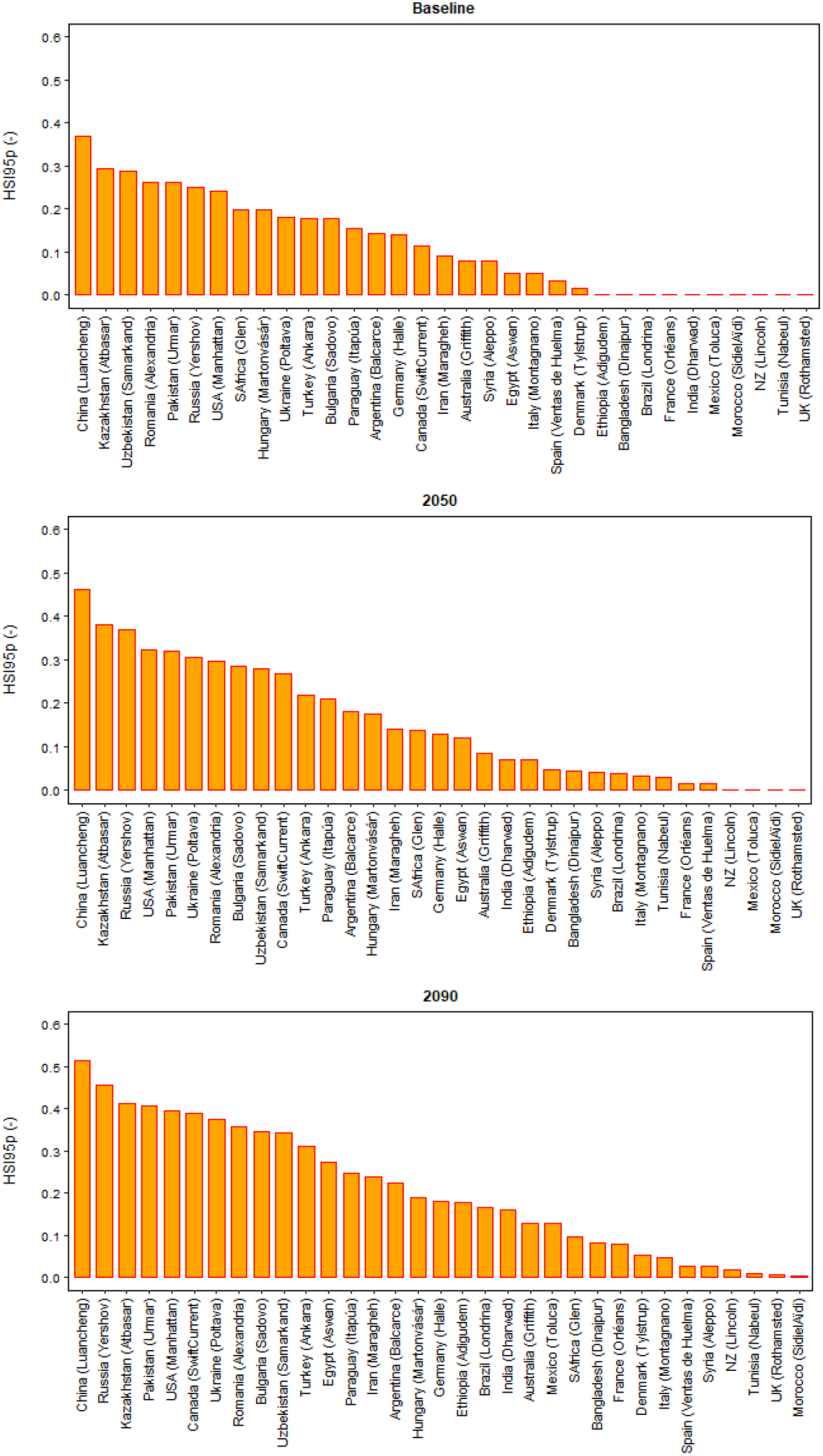
Ranking of 95th percentile Heat Stress Index (HSI95p) as mean over 15 GCMs in different wheat producers under baseline (1985–2015) and future climates (2050 and 2090), based on the extreme sites (see Methods)

Under baseline climate, mean global relative yield loss due to extreme high temperature around flowering was estimated as HSI95p ~ 0.11 (HSI95p is the 95th percentile of the Heat Stress Index, see Methods) (Fig. 5). The estimated HSI95p was highest (> 0.37) in China, whereas it was lowest (~ 0) in UK, France, and New Zealand (Figs. 6 and 7). Among the other major wheat producers and exporters, estimated relative yield loss was moderate (0.10 ≤ HSI95p ≤ 0.30) in Kazakhstan, Romania, Pakistan, Russia, USA, Ukraine, Turkey, Germany and Canada, and low (HSI95p < 0.10) in Australia, India, Iran, Italy, Spain and Mexico.

The global HSI95p was predicted to rise considerably, viz. by 32% and 77% in 2050 and 2090, respectively, compared to the baseline (Fig. 5). The future predicted yield loss due to extreme temperature around flowering increased in almost all the wheat-producing countries and the number of countries substantially affected by extreme heat stress increased with time (Figs. 6 and 7). Among major producers and/or exporters, the highest relative increase in HSI95p under 2050 climate was predicted in Canada (up by 135%), followed by Ukraine, Bulgaria, Russia and Iran. Under 2090 climate, HSI95p was predicted to rise by 243% in Canada, followed by Iran, Ukraine, Russia, Bulgaria, Turkey and USA. In terms of predicted absolute yield loss due to extreme heat stress around flowering, China was predicted to have the highest impacts (HSI95p > 0.40) under near-future climate, followed by Kazakhstan, Russia, USA, Pakistan and Ukraine (HSI95p > 0.30). Similar but higher magnitudes of yield losses, affecting a greater number of countries, were predicted under far-future climate, viz. China (HSI95p > 0.50), followed by Russia, Kazakhstan and Pakistan (HSI95p > 0.40), USA, Ukraine, Romania, Uzbekistan, Turkey and Canada (HSI95p > 0.30).

**Fig. 8:**
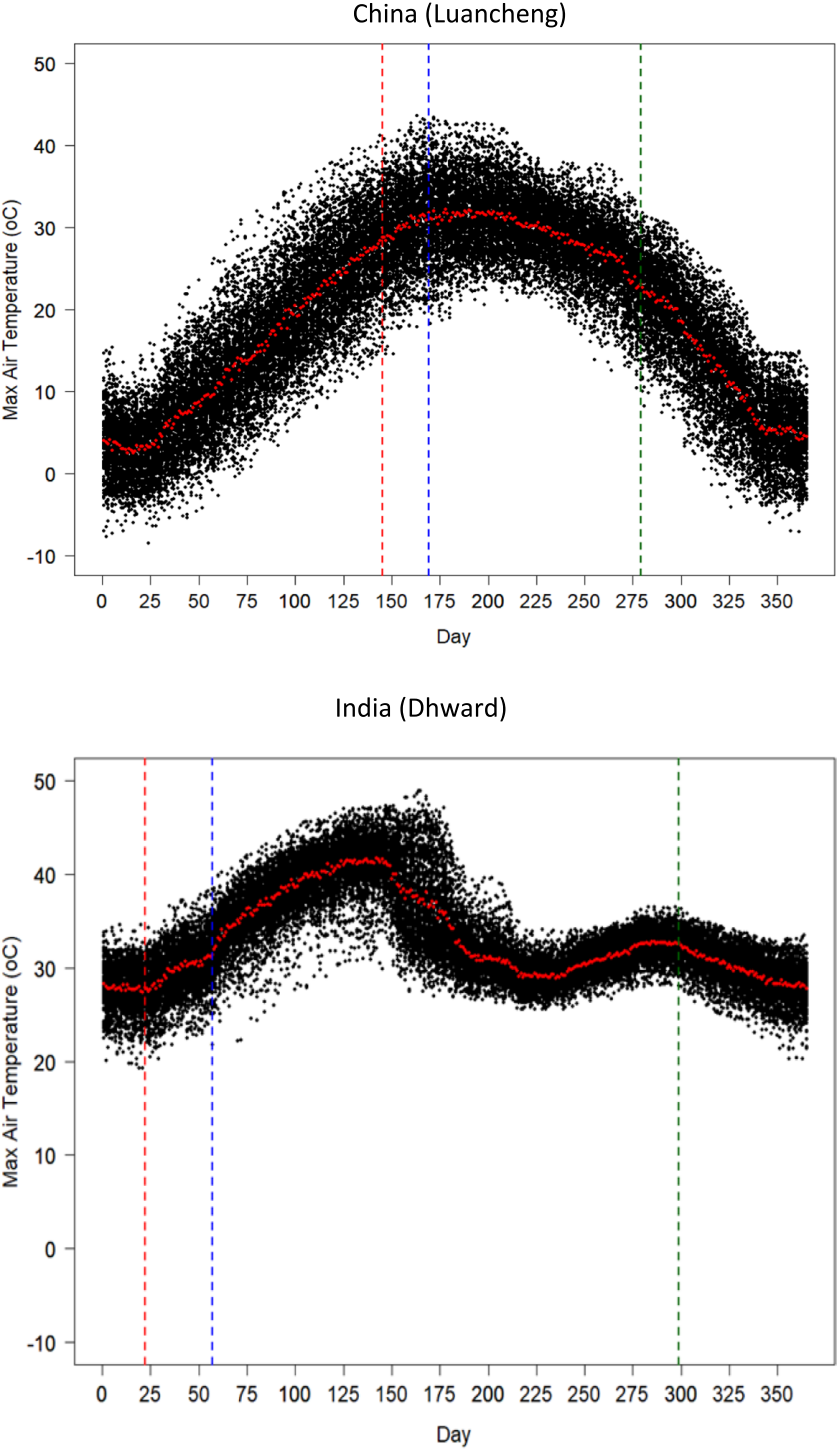
Maximum air temperature during the wheat growing season under baseline climate (1985–2015) in China (Luancheng) and India (Dharwad). The green, red, and blue vertical dash lines represent sowing, flowering and maturity date, respectively, of current local wheat cultivars under baseline climate

Two contrasting trends of heat stress were identified under baseline and future climates, viz. (i) in countries where mean wheat growing season temperature is comparatively low, but heat stress at flowering (HSI95p) is high, such as China, Ukraine and Romania, and (ii) in countries where mean wheat growing season temperature is high, but heat stress at flowering (HSI95p) is moderate, such as India, Bangladesh, Australia, Brazil, Egypt, Ethiopia, Spain and Mexico (Figs. 6 and 7, and Supplementary Table S1 and Fig.S8). Detailed site analysis based on the observed baseline climate in countries in category ‘i’ (e.g., China) revealed that air temperature could exceed critical thresholds during flowering more often, even if the temperature throughout the wheat growing season is relatively low, due to wider variability in the distribution of air temperature (Fig. 8), resulting in higher relative yield loss. On the other hand, the flatter and narrower distribution of air temperature in countries in category ‘ii’ (e.g., India) may result in air temperatures exceeding critical thresholds during flowering less often (Fig. 8), despite the comparatively higher mean wheat season temperature, resulting in lower yield loss. The present study suggests that the potential increase in heat stress under future climates could depend not only on specific wheat growing regions with cooler and hotter wheat growing seasonal temperatures, but also on local conditions, such as favourable or harsh microclimates, that prevail at critical crop phenological stages, resulting from complex interactions within local climate patterns, annual temperature distribution and other environmental factors, as well as crop genotype and management (Riedesel et al. 2024; Zampieri et al. 2017).

Globally and for most of the wheat producers, the significant increases in extreme heat stress around flowering in near- and far-future climates could be linked to a substantial increase in air temperature, both minimum and maximum, during the wheat growing season (Figs. 5 and 6). Rezaei et al. (2015) indicated that accelerated winter wheat phenology due to warmer temperature could almost compensate impact of heat stress under changing climate. However, our results indicate that, despite early flowering, wheat may not be able to escape extreme heat stress around flowering completely in the future climates due to a sharp increase in air temperature (Figs. 5, 6 and 7). With the increasing risk of higher global yield losses with time in near- (2050) and far-future (2090) climate scenarios (Fig. 5), and the increasing number of wheat-producing countries that could be substantially affected by heat stress (Figs. 6 and 7), heat stress at flowering was identified as an emerging threat to global wheat production under future climates. Global hot spots of wheat heat stress have been identified by other studies in different regions, for example in Eastern China, South-Western Russia, Northern United States, southern Canada, Pakistan and Kazakhstan (Teixeira et al. 2013). In a few studies, global heat stress has been considered as an emerging threat to wheat yield under climate change (Liu et al. 2021; Teixeira et al. 2013).

## General discussion and conclusion

Drought is a widespread environmental stress in agriculture and represents a significant challenge for future food security. Hence, enhancing yields in dry environments is a major objective in plant breeding. Under baseline climate, the absolute magnitude of mean global yield loss due to extreme drought around flowering was estimated to be much greater than the yield loss due to extreme temperature around flowering (DSI95p ~ 0.37 compared with HSI95p ~ 0.11). Some experts have predicted that the influence of drought will intensify alongside climate change, underscoring the need to breed drought-resistant crops (Raza et al. 2023; Webber et al. 2018). However, our results indicate that the vulnerability of wheat production to drought stress around flowering may not increase under future climates, mainly due to earlier flowering as a result of warmer temperatures. A few studies reported similar results (Le Roux et al. 2024; Hickey et al. 2022; Shavrukov et al. 2017). In fact, relative yield losses due to extreme drought stress at flowering are likely to decrease across wheat producers globally. That said, it should be noted that yield losses due to severe drought were predicted to remain still high under future climates, with a very high impact (up to DSI95p ~ 0.80) in some important wheat producers, including China, USA, Argentina, Iran, Turkey, Spain and Romania.

In contrast, the potential yield losses due to extreme high temperature around flowering were projected to rise substantially with an increasing trend with time, viz. near- (2050) and far-future (2090). Despite flowering earlier under future climates, wheat may still face more frequent and severe short-term, extreme heat stress at flowering in almost all the wheat-producing countries due to a sharp increase in the growing season air temperature under climate change. The heat stress at flowering was identified as an emerging threat to global wheat production under climate change, substantially increasing the vulnerability of wheat production in many major wheat-producing and/or exporting countries in near- and far-future climates. Some studies have also indicated heat stress as an emerging threat for crop production under climate change in different regions of the world (Farhad et al. 2023; Ortiz-Bobea et al. 2019; Stratonovitch and Semenov 2015).

Among the top wheat producers and/or exporters, drought stress at flowering could be highest under future climates in China, USA, Russia, Turkey, Romania, Argentina, Australia and Kazakhstan, whereas extreme high temperature stress at flowering could be highest in China, Russia, Kazakhstan, USA, Pakistan, Canada, Ukraine, Romania and Turkey. This means that China, USA, Russia, Romania, Turkey, and Kazakhstan will be severely affected by both of these stresses at flowering under climate change. An increasing risk of global climatic wheat yield shock simultaneously in multiple breadbaskets has been observed and/or predicted under future climate change, including China, south Asia, USA, Europe, and Australia (Anderson et al. 2023; Zhu et al. 2021; Gaupp et al. 2019).

In our study, 14 wheat producing countries had more than one study site, while 19 had a single site. To estimate the impacts of extreme heat and drought stresses around flowering at country level, the most extreme site was selected separately for heat and drought stress impacts for those countries with multiple sites. However, our results indicated that the highest heat and drought stresses around flowering would not necessarily occur at the same site or region even within a country under future climate. For example, heat and drought stresses at flowering could be highest at different sites or regions within China (DSI95p at Xuchang and HSI95p at Luancheng), and USA (DSI95p at Lind and HSI95P at Manhattan). On the other hand, both stresses were predicted to be highest at the same sites or regions within Turkey (Ankara), Russia (Yershov) and Kazakhstan (Atbasar). The prevalence of extreme heat and drought stresses in these countries has been reported in other studies in which spatial variation in heat and drought stresses were found to be common as site specific conditions (environment × management × genotype) mostly determine heat as well as drought induced yield losses (Riedesel et al. 2024; Afroz et al. 2023; Hao et al. 2022).

In this study, the impacts of extreme heat and extreme drought events around flowering on grain numbers and grain yields were modelled separately. A few studies have indicated increasing co-occurring extreme hot and dry events during the growing season and a potential non-linear compound impact of extreme heat and drought stresses on crop yield (Heino et al. 2023; Yin et al. 2022). However, the combined effect of heat and drought stresses around flowering was not evaluated in this study, because our objective was to compare the future impacts of extreme heat and extreme drought stresses in order to identify priority target and inform decision-making on wheat genetic adaptation and breeding priorities for developing new cultivars under climate change. Ideally, climate change impact studies should incorporate multiple crop models, but only one wheat model (Sirius) was used here. Sirius 2018 is an advanced, process-based model which is being continually refined and evaluated and has been shown to perform well globally across different management systems and environments (Semenov 2021). The impacts of extreme high temperature and drought events during the short period of reproductive development are difficult to implement in a process-based model due to their complex and short-term in nature, and lack of high-quality experimental data sets needed for model calibration. Very few wheat models incorporated the impacts of extreme heat and extreme drought at flowering, including Sirius (Senapati et al. 2019; Stratonovitch and Semenov 2015). Rather than using an ensemble of wheat models, which are lacking these specific impact mechanisms, only Sirius was used, and different associated crop eco-physiological processes were investigated in detail. All the current wheat varieties used in the study were assumed to be equally susceptible to short-term, but extreme high temperature or extreme drought stresses around flowering (Supplementary Figs. S2-S4). A few current varieties may have some degree of tolerance to these stresses, but, quantifying the magnitude of sensitivity to extreme heat or drought stresses around flowering for each individual variety is difficult due to lack of experimental data. The cultivar parameters related to extreme heat stress or drought stress around flowering, as used in this study, were found reasonable, although they were not cultivar or site specific (Senapati et al. 2021, 2019; Semenov 2021; Stratonovitch and Semenov 2015). Further calibration and investigation for individual cultivar and site require dedicated field experiments and new high quality data sets. Different Shared Socioeconomic Pathways (SSPs) have been constructed, based on different assumptions of future GHG emissions along with different economic development scenarios (Riahi et al. 2017; O'Neill et al. 2016). Climate change impacts preferably need to be assessed across different SSPs. However, as main aim of our study was to assess the impacts of extreme high temperature and drought events around flowering, only the highest GHG emission or worst climate change scenario (SSP5-8.5) was selected. Similarly, only rainfed wheat was investigated while irrigated wheat systems were not considered, reflecting the dominance of rainfed wheat in global wheat production systems (Dadrasi et al. 2023b; Portmann et al. 2010). The application of irrigation could reduce or even eliminate drought stress, depending on the availability, timing and quantity of irrigation. It may also reduce heat stress to some extent through evaporative cooling (Siebert et al. 2017). Additionally, any future agronomic management adaptation, which could potentially reduce impacts or contribute to tolerance or avoidance of heat and drought stresses at flowering under climate change, such as early sowing (Paudel et al. 2023; Hunt et al. 2019), was not investigated.

Despite the above limitations, this study estimated the potential magnitude of impacts of extreme high temperature and drought events around flowering on wheat yield globally under future climate change scenarios, using a state-of-the-art wheat model (Sirius) and climate scenarios based on the latest CMIP6 ensemble. Using an ensemble of GCMs enabled us to estimate the uncertainty in the impacts arising from different climate projections, propagated through different GCMs. The major finding of the study is that global drought stress at flowering will decrease in near- and far-future climates, although it will remain high, whereas heat stress at flowering will increase significantly, with the magnitude of the impact of heat stress at flowering approaching that of drought stress with time. Heat stress at flowering was, therefore, identified as an emerging threat to wheat yield, substantially increasing the vulnerability of wheat under future climate change. The study also identified the major wheat producers and/or exporters that are likely to be highly affected by short-term, extreme temperature and drought events around flowering in future climates. Building tolerance to short-term, but extreme high temperature and drought stresses at flowering must become a target for wheat breeders. These results should be acted upon now by breeders to reduce global wheat yield vulnerability to the short-term, low frequent, but high impact, extreme climate events predicted under climate change and protect our food security.

The interconnected nature of the global food supply chain means that disruptions in one region can have effects on food supply and food insecurity worldwide. Mitigating these threats may necessitate a holistic approach that includes adopting improved resource management, conservation, and sustainable agricultural practices, alongside the development and deployment of new wheat cultivars tolerant to short-term, but extreme heat and drought stresses around flowering.

## Supplementary Information

Below is the link to the electronic supplementary material.

Supplementary file1 (PDF 1.76 MB)

## Data Availability

All data supporting the findings of this study are included within the article and its supplementary information. Any further information regarding this study is available from the corresponding authors on request.

## Code availability

The Sirius model and the stochastic weather generator LARS-WG used in the present study are available from https://sites.google.com/view/sirius-wheat/ (or 10.5281/zenodo.4572624) and https://sites.google.com/view/lars-wg/ (or 10.5281/zenodo.4572752), respectively.
