# Supplementary material for: Extreme heat and drought at flowering could threaten global wheat yields under climate change
Source: Clim Change. 2026 Jan 29;179(2):28. doi: 10.1007/s10584-025-04054-8 (PMC12855381; doi:10.1007/s10584-025-04054-8)
Supplement: Supplementary file 1 — Supplementary file1 (PDF 1.76 MB) [file 10584_2025_4054_MOESM1_ESM.pdf]

Extreme heat and drought at flowering could threaten global wheat yields under climate change

Supplementary Information

Supplementary Methods
Supplementary Tables S1-S3
Supplementary Figures S1-S10.

Supplementary Methods

1 Sirius model

Sirius is a process-based, eco-physiological, advanced wheat model, consisting of different sub-models that describe soil, climate, crop management and plant processes (Semenov 2021; Senapati et al. 2019; Stratonovitch and Semenov 2015; Jamieson et al. 2007; Lawless et al. 2005; Brooks et al. 2001; Jamieson and Semenov 2000; Jamieson et al. 1998b; Jamieson et al. 1998a). The detailed plant processes in Sirius include water and nitrogen dynamics, photosynthates production, partitioning of photosynthates into leaf, stem, grain and root, and phenological developments, along with responses to limitations of water and N supply, including the impacts of adverse, short-term climatic events such as heat and drought stress. Sirius runs on a daily time-scale and requires daily weather data (minimum and maximum air temperatures, rainfall, solar radiation etc.), soil descriptions (soil water characteristics, and soil organic and mineral nitrogen contents of different layers), soil and crop management (sowing date and rate, date and rate of fertiliser and irrigation applications etc.), and cultivar information (Phyllochron, daylength and vernalisation responses, potential maximum leaf number and size, grain filling duration, anthesis and maturity dates etc.) as inputs.

1.1 Photosynthesis and biomass accumulation

Photosynthesis and biomass production are simulated daily as the product of radiation use efficiency (*RUE*) and intercepted photosynthetically active radiation (*PAR*), limited by water, temperature and nitrogen stresses. The quantity of intercepted radiation depends on leaf area and the 'light extinction coefficient'. The shortage of N limits leaf area, and hence light interception and biomass production.

1.2 Leaf and canopy development

Leaf area development in each layer is simulated by a thermal time sub-model, and actual leaf area is calculated using a simple limitation rule. Canopy development is simulated as a series of leaf layers associated with individual mainstem leaves. Final leaf numbers are determined by a combined response to day length and vernalisation. Maximum area of flag leaf (*A*) influences the rate of canopy expansion and the maximum achievable LAI.

1.3 Phenology and grain development

Wheat phenological development is linked to the mainstem leaf appearance rate (phyllochron: P_h), day length response (P_p), 'vernalization responses', and 'duration of grain filling' (*Gf*). P_h is the thermal time required for the appearance of successive leaves and G_f is cultivar-specific amount of thermal time requires to complete grain filling. *Ph, Pp and* G_f control the rate of crop development, flowering date and maturity. During grain filling, assimilates for the grain are available from two sources viz. new photosynthates produced from intercepted radiation after anthesis and labile carbohydrates stored mostly in the stem before anthesis.

1.4 Root growth and soil water uptake

Soil is described as a cascade of 5 cm layers up to a user-defined depth. Roots continue to grow until reaching a soil-dependent maximum depth or until anthesis, whichever occurs first. Each soil layer contains root available water (RAW) (|water potential| < 1.5 MPa) and unavailable water (|water potential| > 1.5 MPa), depending on its water retention characteristics. Only a proportion of the available soil water can be extracted by plants from each layer of the root zone on any day, depending on the 'efficiency of water extraction' (λ) and 'rate of root water uptake' (R_u).

1.5 Leaf senescence

Leaf senescence is expressed in thermal time and linked to the rank of the leaf in the canopy. Total canopy senescence synchronizes with the end of grain filling. Water, heat and N stresses could accelerate leaf senescence, reduce leaf area, and adversely affect biomass assimilation, phenological development, grain filling and yield.

1.6 Sink potential

In the absence of extreme heat and drought events around flowering, potential sink capacity of grains (Y_{pot}, g m^{-2}) is set in Sirius as the product of the ear dry mass accumulated prior to anthesis (DM_{ear}, g m^{-2}), potential primary grain setting number per unit of ear dry mass (N_{pot}, grains g^{-1}) and potential weight of a single grain (W_{pot}, g $grain^{-1}$):

$$Y_{pot} = DM_{ear} \times N_{pot} \times W_{pot}$$

In the absence of abiotic stress around flowering, a sufficiently large sink capacity (N_{pot} =100 grains g^{-1} and W_{pot} = 0.050 g $grain^{-1}$) is set to accommodate newly produced as well as re-translocatable photosynthate reserves. Therefore, grain yield is determined by the source capacity of the crop or crop cultivar in the absence of abiotic stress.

However, in the presence of short but extreme heat and drought stresses around flowering, Y_{pot} is decreased through reduction of N_{pot} and W_{pot} to actual primary fertile grain setting number (N) and actual weight of an individual grain (W), respectively.

1.7 Impact of water limitation over entire growing period

In Sirius, water stress throughout the crop growing period adversely affects both source (photosynthate production) and sink strength (grain filling). Photosynthate production is reduced by water limitation and new crop biomass production decreases proportionally to 'response of photosynthesis to water stress' (W_{SA}), defined as $W_{SA} = SF_{\beta}^{\beta}$, where SF is a stress factor and β is a cultivar-independent constant. The rate of leaf senescence increases under water limitation by a factor, 'water stress senescence maximum factor' (W_{SSAmax}), that modifies the daily increment of thermal time. Earlier leaf senescence will reduce grain yield by decreasing grain size due not only to a reduction in intercepted radiation and photosynthesis, but also to a decrease in translocation of the labile crop reserve carbohydrate to the grain due to premature termination of grain filling driven by early leaf senescence.

1.8 Impact of temperature over entire growing period

High temperatures (maximum temperature > 34 °C) during the crop growing period could accelerate phenological development as well as leaf senescence in wheat. In Sirius, higher temperatures would accelerate the rate of phenological development by meeting the thermal time requirements of each phenological development stage more rapidly. To account for the impact of high temperature on leaf senescence, a daily 'leaf thermal time increment', ΔT (°C), is calculated as the average of the sum of the 3-hourly temperatures above a base temperature, T_b = 0 °C. Hourly temperature is interpolated by a sine curve using daily maximum and minimum temperatures. The 3-hourly temperatures, T_i, are multiplied by an 'accelerated leaf senescence factor', R_i^L (dimensionless), to account for the acceleration of leaf senescence caused by high temperature:

$$\Delta T = \sum_{n=1}^{8} \text{Max}\,(0, (R_i^L \times (T_i - T_b)))/8.$$

R_i^L increases linearly from 1 when T_i exceeds a temperature threshold ('heat stress leaf senescence increases threshold temperature': T^L, °C) as $R_i^L = 1 + \max(0, T_i - T^L) \times S^L$, where S^L (°C^{-1}) is the slope of the senescence acceleration per unit of canopy temperature above T^L. Early leaf senescence due to high temperature reduces the total leaf area, and thus the amount of light interception and photosynthate production. Grain filling may also end prematurely if the canopy is fully senesced.

1.9 Sirius's calibration and validation

Sirius has been extensively calibrated and validated over the last three decades across the world for modern wheat cultivars, as used in this study (Supplementary Tables S1 and S2), in AgMIP, BBSRC and MACSUR studies, and has performed well under diverse climatic conditions, including Free-Air CO_2 Enrichment experiments (Guarin et al. 2022; Asseng et al. 2019; Liu et al. 2019; Wang et al. 2017; Liu et al. 2016; Asseng et al. 2015; Martre et al. 2015; Stratonovitch and Semenov 2015; Asseng et al. 2013). Sirius's estimated potential yield of current wheat cultivars in rainfed condition under baseline climate in the present study was compared with the similar estimates by GYGA (GYGA 2024), found significant agreement (Supplementary Fig. S9, correlation coefficient, r = 0.78*, mean difference, M = 0.16 t ha^{-1}). In addition to these background cultivar parameters (Supplementary Tables S1 and S2), a similar set of parameters associated only with extreme heat or drought stress impacts around flowering was used for all current cultivars-sites (Supplementary Figs. 2-4). Model calibrations for individual cultivar and individual site were not possible due to the lack of dedicated and high-quality field experiments, hence assumed equally susceptible to short-term, but extreme high temperature and extreme drought stresses around flowering.

Supplementary Tables

Table S1 Site characteristics of 53-study sites across the global wheat growing regions.

Country	Site	Site No.	Latitude	Longitude	SAW[a] (mm)	Growing season Temp[b] (°C)	Growing season Prec[c] (mm)	Cultivar[d]	Growth habit	Sowing date
Argentina	Balcarce	S1	-37.75	-58.30	176.3	14.2	343	Oasis	Spring	5-Aug
Argentina	Santa Rosa	S2	-36.37	-64.17	180.9	12.7	225	Avocet	Spring	5-Jun
Australia	Griffith	S3	-34.17	146.03	176.3	13.4	170	Avocet	Spring	15-Jun
Australia	Merredin	S4	-31.50	118.20	167.7	13.4	180	Wyalkatchem	Spring	24-May
Bangladesh	Dinajpur	S5	25.65	88.68	176.3	20.5	36	Kanchan	Spring	1-Dec
Brazil	Londrina	S6	-23.31	-51.13	176.3	18.4	340	Atilla	Spring	20-Apr
Bulgaria	Sadovo	S7	42.13	24.93	125.9	9.6	386	Brigadier	Winter	15-Oct
Canada	Josephsburg	S8	53.70	-113.06	159.1	14.8	254	Steklov-24	Spring	4-Jun
Canada	Swift Current	S9	50.28	-107.78	153.7	17.4	168	Steklov-24	Spring	2-Jun
China	Dingxi	S10	35.46	104.73	243.2	15.1	271	Pishtaz	Spring	18-Apr
China	Luancheng	S11	37.53	114.41	176.3	10.1	186	SM15	Winter	5-Oct
China	Nanjing	S12	32.03	118.48	176.3	12.8	622	NM13	Winter	5-Oct
China	Xuchang	S13	34.01	113.51	174.8	11.3	211	Wenmai-6	Winter	10-Dec
Denmark	Tylstrup	S14	57.20	9.90	177.0	6.8	580	Avalon	Winter	18-Oct
Egypt	Aswan	S15	24.10	32.90	176.3	18.7	1	Seri-M-82	Spring	20-Nov
Ethiopia	Adi Gudem	S16	13.25	39.51	170.1	20.8	434	Debeira	Spring	6-Jul
France	Clermont-Ferrand	S17	45.80	3.10	177.0	9.4	355	Thesee	Winter	15-Nov
France	Estrées-Mons	S18	49.88	3.00	176.3	9.1	538	Bermude	Winter	5-Oct
France	Orléans	S19	47.83	1.91	176.3	9.3	474	Apache	Winter	20-Oct
Germany	Halle	S20	51.51	11.95	177.0	8.2	393	Claire	Winter	20-Oct
Germany	Schleswig	S21	54.53	9.55	176.3	7.7	766	Dekan	Winter	25-Sep
Hungary	Martonvásár	S22	47.35	18.81	191.8	8.2	350	Apache	Winter	15-Nov
India	Dharwad	S23	15.43	75.12	176.3	24.4	76	Debeira	Spring	25-Oct
India	Indore	S24	22.72	75.86	176.3	21.7	39	HI-1544	Spring	25-Oct
India	Ludhiana	S25	30.90	75.85	176.3	17.1	87	HD-2687	Spring	15-Nov
Iran	Maragheh	S26	37.38	46.23	217.8	7.9	244	SM15	Winter	13-Nov
Iran	Quchan	S27	37.12	58.47	209.9	5.3	250	Pishtaz	Spring	24-Oct
Italy	Montagnano	S28	43.30	11.80	177.0	10.3	451	Creso (Durum)	Winter	25-Nov
Italy	Policoro	S29	40.20	16.66	110.7	12.6	291	Basri-Bey	Spring	28-Nov
Kazakhstan	Atbasar	S30	52.33	68.58	179.2	14.2	120	Steklov-24	Spring	10-Apr
Kazakhstan	Karagandy	S31	50.17	72.74	176.3	13.5	139	Steklov-24	Spring	20-May
Mexico	Toluca	S32	19.40	-99.68	176.3	14.6	652	Tacupeto-C2001	Spring	10-May
Morocco	Sidi el Aïdi	S33	33.07	-7.00	166.9	13.2	277	Yecora-T48	Spring	05-Nov
New Zealand	Lincoln	S34	-43.62	172.18	270.0	10.3	448	Claire	Winter	20-Apr
Pakistan	Urmar	S35	34.00	71.55	184.6	18.4	234	Yecora	Spring	15-Jan
Paraguay	Itapúa	S36	-26.79	-55.67	221.8	17.3	476	Atilla	Spring	25-May
Romania	Alexandria	S37	43.98	25.35	197.0	8.2	352	Brigadier	Winter	21-Oct
Russia	Krasnodar	S38	45.02	38.95	176.3	10.8	606	Brigadier	Winter	15-Sep
Russia	Yershov	S39	51.36	48.26	206.1	19.7	100	Steklov-24	Spring	06-May
S Africa	Glen	S40	-28.95	26.33	134.8	13.2	95	Wyalkatchem	Spring	04-Jun
Spain	Lleida	S41	41.63	0.60	177.0	11.5	193	Creso (Durum)	Winter	25-Nov
Spain	Ventas de Huelma	S42	37.16	-3.83	131.4	15.8	131	Basri-Bey	Spring	18-Feb
Syria	Aleppo	S43	36.01	36.56	115.9	12.6	443	Pishtaz	Spring	22-Nov
Tunisia	Nabeul	S44	36.75	10.75	170.3	13.5	284	Pishtaz	Spring	1-Dec
Turkey	Ankara	S45	39.92	32.85	188.0	8.3	365	Fuller	Winter	1-Nov
Turkey	Izmir	S46	38.60	27.06	176.3	11.9	561	Basri-bey	Spring	15-Nov
UK	Rothamsted	S47	51.82	-0.37	176.3	8.6	514	Avalon	Winter	15-Oct
Ukraine	Poltava	S48	49.37	33.17	176.3	6.9	433	Brigadier	Winter	15-Sep
USA	Lind, WA	S49	47.00	-118.56	140.9	8.8	237	AC-Radient	Winter	14-Sep
USA	Madison, WI	S50	43.03	-89.40	176.3	6.8	699	Brigadier	Winter	15-Sep
USA	Manhattan, KS	S51	39.14	-96.63	176.3	10.2	565	Fuller	Winter	1-Oct
USA	Watkinsville, GA	S52	34.03	-83.41	112.9	12.9	721	Brigadier	Winter	25-Nov
Uzbekistan	Samarkand	S53	39.70	66.98	168.1	11.4	281	SM15	Winter	14-Dec

SAW[a]: Soil available water capacity.
Temp[b]: Average air temperature during wheat growing season (sowing-maturity)
Prec[c]: Average cumulative precipitation during wheat growing season (sowing-maturity)
Cultivar[d]: Detailed characteristics of the current local cultivars are provided in Table S2

Table S2 Characteristics of current local wheat cultivars, as used in this study, as calibrated and validated in the Sirius wheat model in AgMIP, BBSRC and MACSUR studies(Guarin et al. 2022; Senapati et al. 2022; Senapati et al. 2021; Senapati et al. 2019; Semenov 2021; Semenov et al. 2014; Asseng et al. 2019; Asseng et al. 2015; Asseng et al. 2013; Liu et al. 2019; Liu et al. 2016; Wang et al. 2017; Martre et al. 2015; Stratonovitch and Semenov 2015).

**Current cultivar**†	**Growth habit**	**Cultivar parameter in Sirius**						
		T_{SOWEM} (ºC day)	T_{ANBGF} (ºC day)	T_{BGEG} (ºC day)	T_{EGFMAT} (ºC day)	P_h (ºC day)	P_p (Leaf h^{-1} day)	A_{max} (m^2 $leaf^{-1}$)
AC_radient	Winter	150.0	50.0	650.0	200.0	90.0	0.6500	0.0065
Apache	Winter	150.0	50.0	650.0	200.0	90.0	0.6500	0.0065
Atilla	Spring	118.6	109.4	750.0	100.0	86.5	1.0380	0.0038
Avalon	Winter	150.0	50.0	650.0	200.0	90.0	0.6500	0.0065
Avocet	Spring	118.6	109.4	750.0	100.0	86.5	1.0380	0.0038
Basri_bey	Spring	118.6	109.4	750.0	100.0	86.5	1.0380	0.0038
Bermude	Winter	150.0	50.0	650.0	200.0	90.0	0.6500	0.0065
Brigadier	Winter	150.0	50.0	650.0	200.0	90.0	0.6500	0.0065
CLAIRE	Winter	150.0	100.0	650.0	200.0	110.0	0.5000	0.0070
CRESO	Durum	160.0	100.0	650.0	200.0	90.0	0.6000	0.0030
Debeira	Spring	118.6	109.4	750.0	100.0	86.5	1.0380	0.0038
Dekan	Winter	150.0	50.0	650.0	200.0	90.0	0.6500	0.0065
Fuller	Winter	150.0	50.0	650.0	200.0	90.0	0.6500	0.0065
HD_2687	Spring	118.6	109.4	750.0	100.0	86.5	1.0380	0.0038
HI_1544	Spring	150.0	100.0	650.0	200.0	120.0	0.6830	0.0040
Kanchan	Spring	118.6	109.4	750.0	100.0	86.5	1.0380	0.0038
NM13	Winter	150.0	50.0	650.0	200.0	90.0	0.6500	0.0065
Oasis	Winter	150.0	100.0	500.0	150.0	85.0	0.5500	0.0035
Pishtaz	Spring	118.6	109.4	750.0	100.0	86.5	1.0380	0.0038
Seri_m_82	Spring	118.6	109.4	750.0	100.0	86.5	1.0380	0.0038
SM15	Winter	150.0	50.0	650.0	200.0	90.0	0.6500	0.0065
Steklov_24	Spring	150.0	50.0	500.0	100.0	85.0	0.5500	0.0035
Tacupeto_c2001	Spring	118.6	109.4	750.0	100.0	86.5	1.0380	0.0038
Thesee	Winter	175.0	100.0	650.0	200.0	94.0	0.4000	0.0040
Wenmai_6	Winter	150.0	50.0	650.0	200.0	90.0	0.6500	0.0065
Wyalkatchem	Spring	118.6	109.4	750.0	100.0	86.5	1.0380	0.0038
Yecora	Spring	118.6	109.4	750.0	100.0	86.5	1.0380	0.0038
Yecora_T48	Spring	118.6	109.4	750.0	100.0	86.5	1.0380	0.0038

† : Current local wheat cultivars, site information of cultivars can be found in Table S1
T_{SOWEM} : Thermal time from sowing to emergence
T_{ANBGF} : Thermal time from anthesis to beginning of grain fill
T_{BGEG} : Thermal time from beginning of grain fill to end of grain fill
T_{EGFMAT} : Thermal time from end of grain fill to harvest maturity
P_h : Phyllochron
P_p : Day length response
A_{max} : Maximum area of flag leaf

Table S3 The 15 Global Climate Models (GCMs) used in the present study from the Coupled Model Intercomparison Project Phase 6 (CMIP6) ensemble.

No.	GCM	Research centre	Country	Grid resolution: latitude x longitude	Reference
1	ACCESS-ESM1-5	Commonwealth Scientific and Industrial Research Organisation (CSIRO)	Australia	1.25° x 1.875°	(Ziehn et al. 2019)
2	BCC-CSM2-MR	Beijing Climate Center (BCC)	China	1.12° x 1.125°	(Wu et al. 2018)
3	CanESM5	Canadian Centre for Climate Modelling and Analysis, Environment and Climate Change Canada (CCCma)	Canada	2.77° x 2.81°	(Swart et al. 2019)
4	CESM2	National Center for Atmospheric Research, Climate and Global Dynamics Laboratory (NCAR)	USA	0.94° x 1.25°	(Danabasoglu 2019)
5	CMCC-ESM2	Fondazione Centro Euro-Mediterraneo sui Cambiamenti Climatici (CMCC)	Italy	0.94° x 1.25°	(Peano et al. 2020)
6	CNRM-CM6-1	Centre National de Recherches Meteorologiques (CNRM), Centre Europeen de Recherche et de Formation Avancee en Calcul Scientifique (CERFACS)	France	1.40° x 1.406°	(Voldoire 2018)
7	GFDL-ESM4	National Oceanic and Atmospheric Administration, Geophysical Fluid Dynamics Laboratory (NOAA-GFDL)	USA	1.00° x 1.25°	(Krasting et al. 2018)
8	GISS-E2-1-G	Goddard Institute for Space Studies (NASA-GISS)	USA	2.00° x 2.50°	(Nasa Goddard Institute for Space Studies 2018)
9	HadGEM3-GC31-LL	UK Met Office Hadley Centre (MOHC)	UK	1.25° x 1.88°	(Roberts 2017)
10	INM-CM5-0	Institute for Numerical Mathematics, Russian Academy of Science (INM)	Russia	1.50° x 2.00°	(Volodin et al. 2019)
11	MIROC6	Japan Agency for Marine-Earth Science and Technology (JAMSTEC), Atmosphere and Ocean Research Institute, The University of Tokyo (AORI), National Institute for Environmental Studies (NIES), and RIKEN Centre for Computational Science (R-CCS, MIROC)	Japan	1.39° x 1.406°	(Shiogama et al. 2019)
12	MPI-ESM1-2-LR	Max Planck Institute for Meteorology (MPI-M)	Germany	1.39° x 1.41°	(Wieners et al. 2019)
13	MRI-ESM2-0	Meteorological Research Institute (MRI)	Japan	1.113° x 1.125°	(Yukimoto et al. 2019)
14	TaiESM1	Research Centre for Environmental Changes, Academia Sinica (AS-RCEC)	Taiwan	0.942° x 1.25°	(Lee and Liang 2020)
15	UKESM1-0-LL	UK Met Office Hadley Centre (MOHC)	UK	1.25° x 1.88°	(Tang et al. 2019)

Supplementary Figures

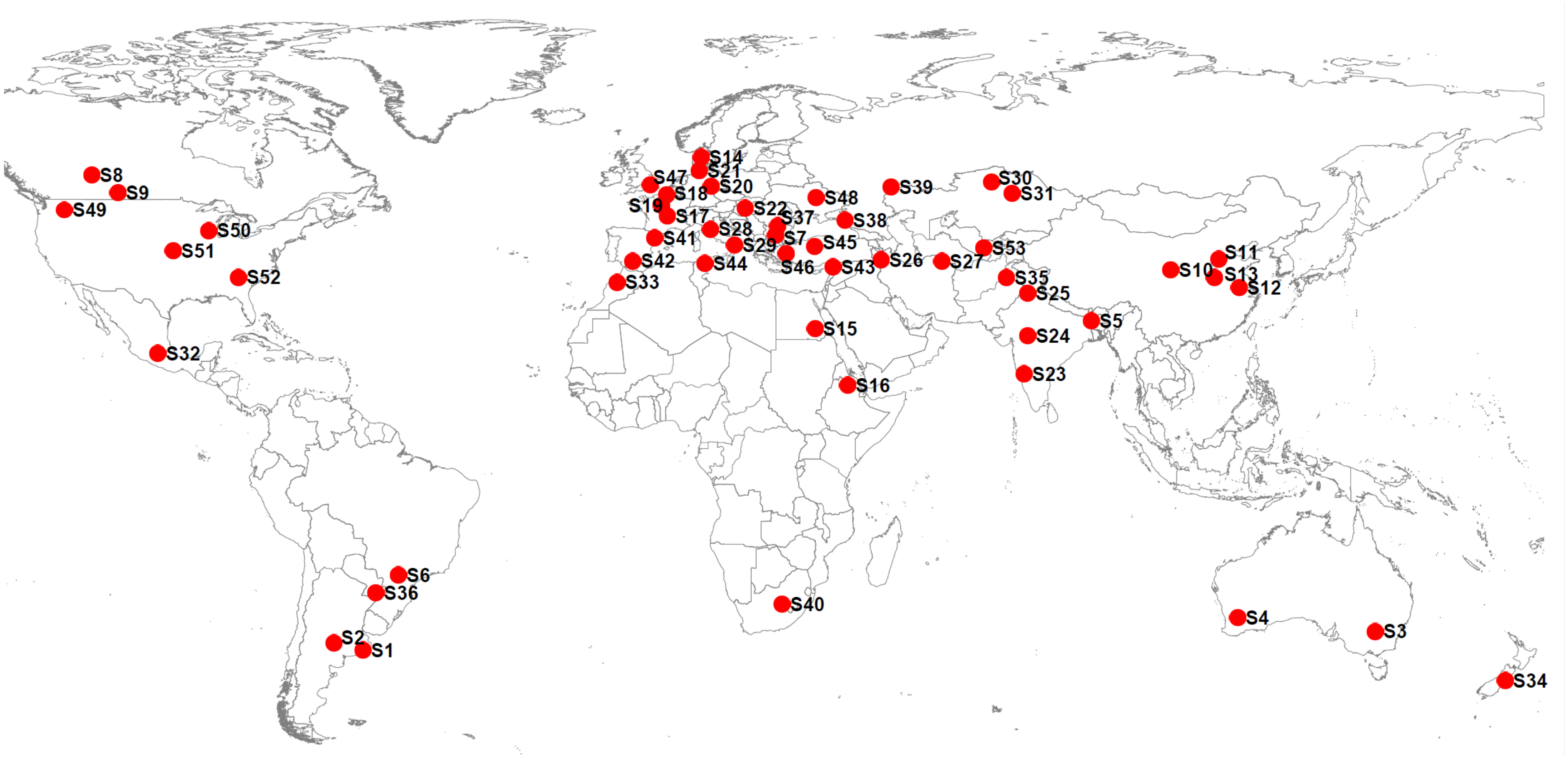

Fig. S1 Study sites. A total of 53 representative study sites selected from 33 wheat-growing countries, covering almost all the global wheat growing environments and the major producers, representing about 91% of current global wheat-growing area and grain production (Senapati et al. 2022; FAOSTAT 2021).

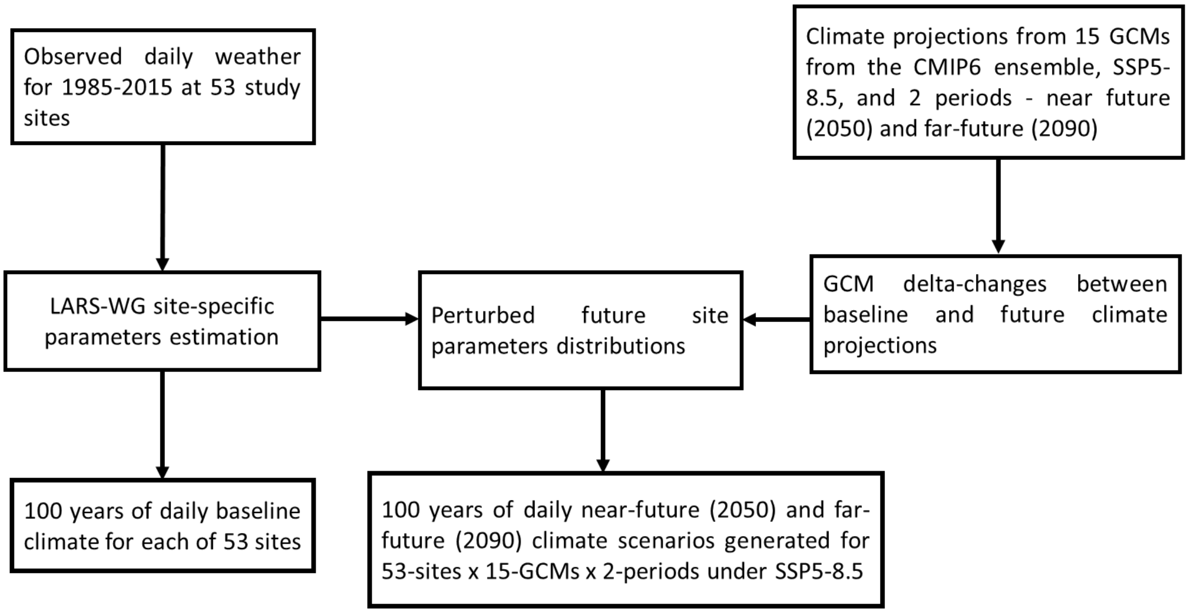

Fig. S2 A flow chart diagram for generation of baseline, and future climate scenarios based on 15 GCMs from the CMIP6 under extreme emission scenarios SSP5-8.5.

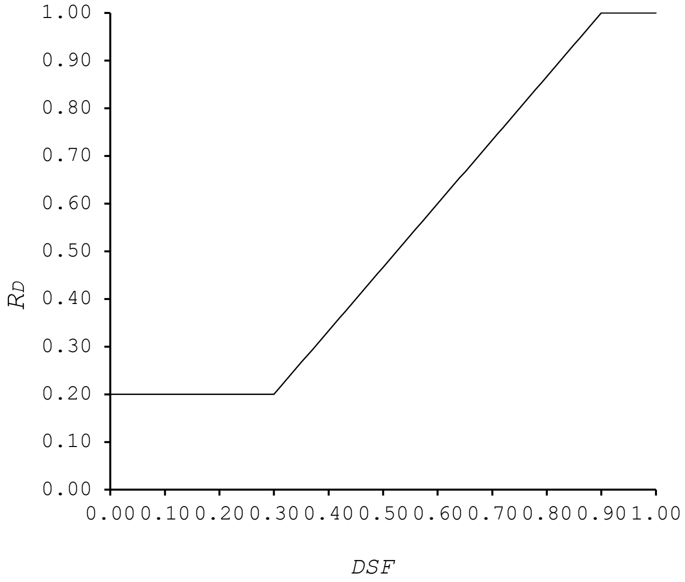

Fig. S3 Relationship between 'Drought reduction factor' of primary fertile grain number set, (R_D, dimensionless) and 'drought stress factor' (*DSF*, dimensionless) during on an average for 15 days around flowering, viz. from 10 d before to 5 d after the flowering date (see Methods). Sirius's parameter values used for cultivars sensitive to short-term, but extreme drought events around flowering as 'drought stress grain number reduction threshold' DSGNT (-): 0.90, 'drought stress grain number reduction saturation' DSGNS (-): 0.30, 'maximum drought stress grain number reduction' DSGNRMax (-): 0.20 (Semenov 2021; Senapati et al. 2021; Senapati et al. 2019).

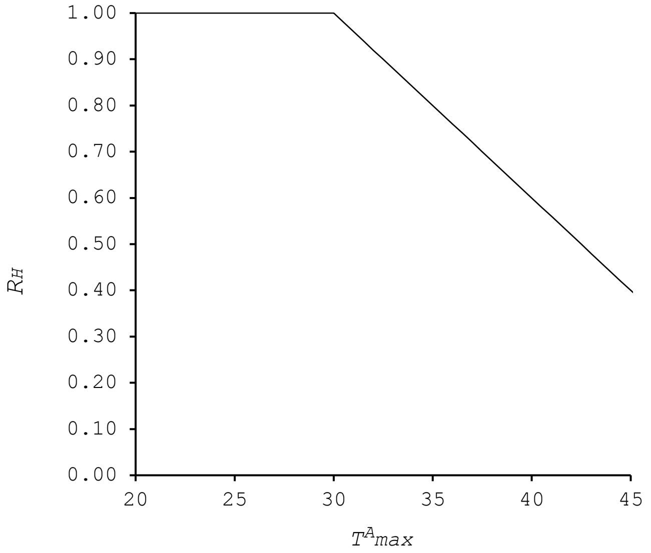

Fig. S4 Relationship between 'heat reduction factor' of primary fertile grain number set, (R_H, dimensionless) and 'maximum canopy temperature', $T^A{}_{max}$ (°C) during a period from 10 days before anthesis to anthesis, which coincides with meiosis and fertilization, when $T^A{}_{max}$ exceeds a 'threshold temperature', T^N (°C) (see Methods). Sirius's parameter values used for cultivars sensitive to short-term extreme heat around flowering as heat stress grain number reduction threshold temperature T^N (°C): 30, heat stress grain number reduction rate S^N (°C^{-1}): 0.04 (Semenov 2021; Senapati et al. 2021; Stratonovitch and Semenov 2015).

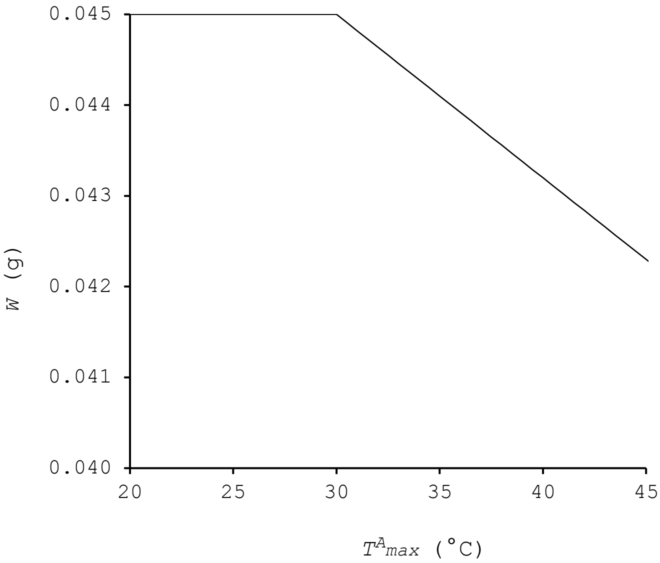

Fig. S5 Relationship between actual individual grain weight (*W*, g) and 'maximum canopy temperature', T^{A}_{max} (°C) at the beginning of grain filling; i.e., a period from 5–12 days after anthesis, when T^{A}_{max} exceeds a 'threshold temperature', T^{W} (°C) (see Methods). Sirius's parameter values used for cultivars sensitive to short-term extreme heat around flowering as heat stress grain weight reduction threshold temperature T^{W} (°C): 30, heat stress grain weight reduction rate S^{W} (°C^{-1}): 0.004 (Semenov 2021; Senapati et al. 2021; Stratonovitch and Semenov 2015).

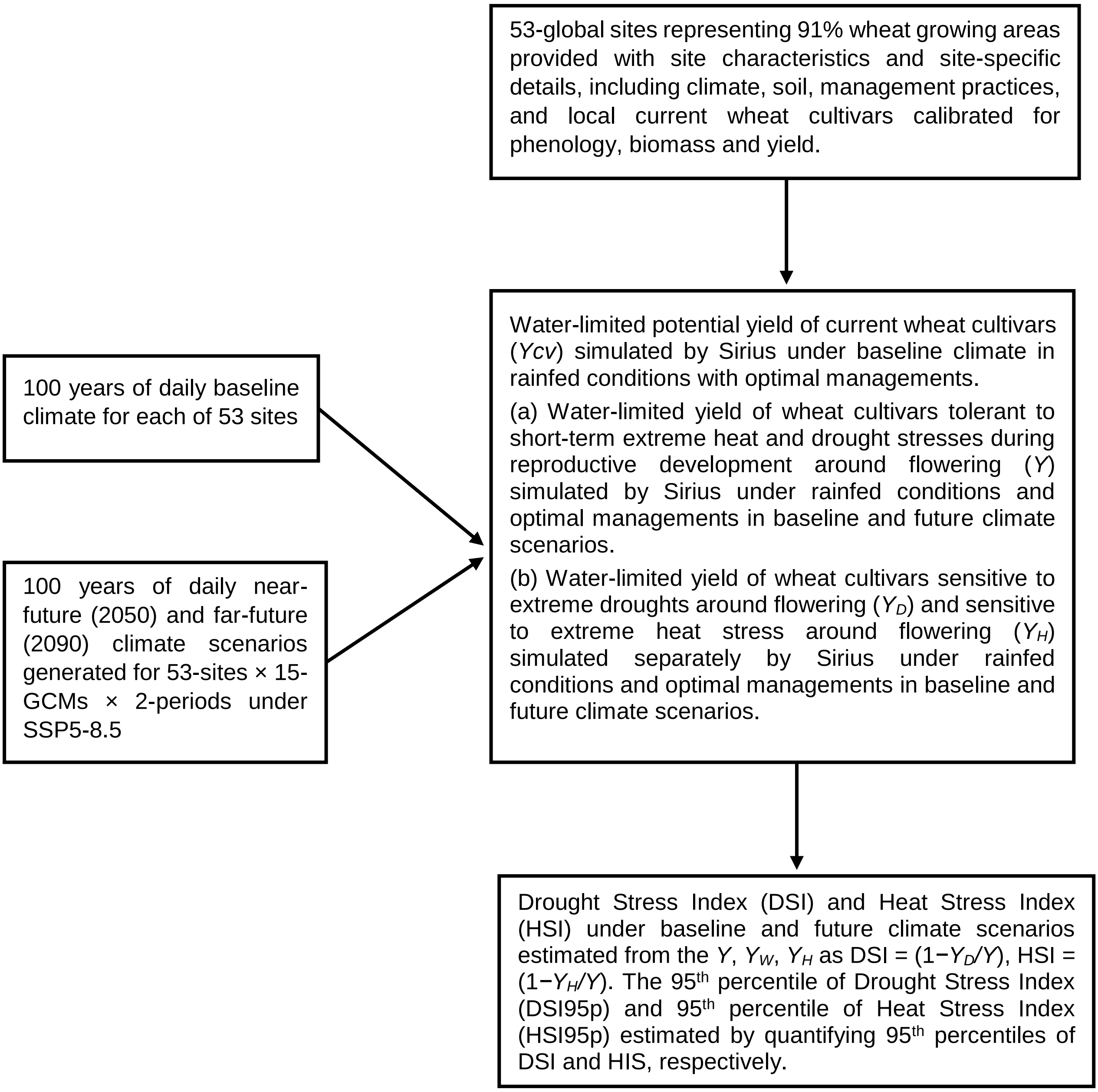

Fig. S6 A workflow diagram for estimation of potential yield of current wheat cultivars (*Ycv*) in rainfed conditions, and 95th percentile of Drought Stress Index (DSI95p) and 95th percentile of Heat Stress Index (HSI95p) under baseline and future climate scenarios.

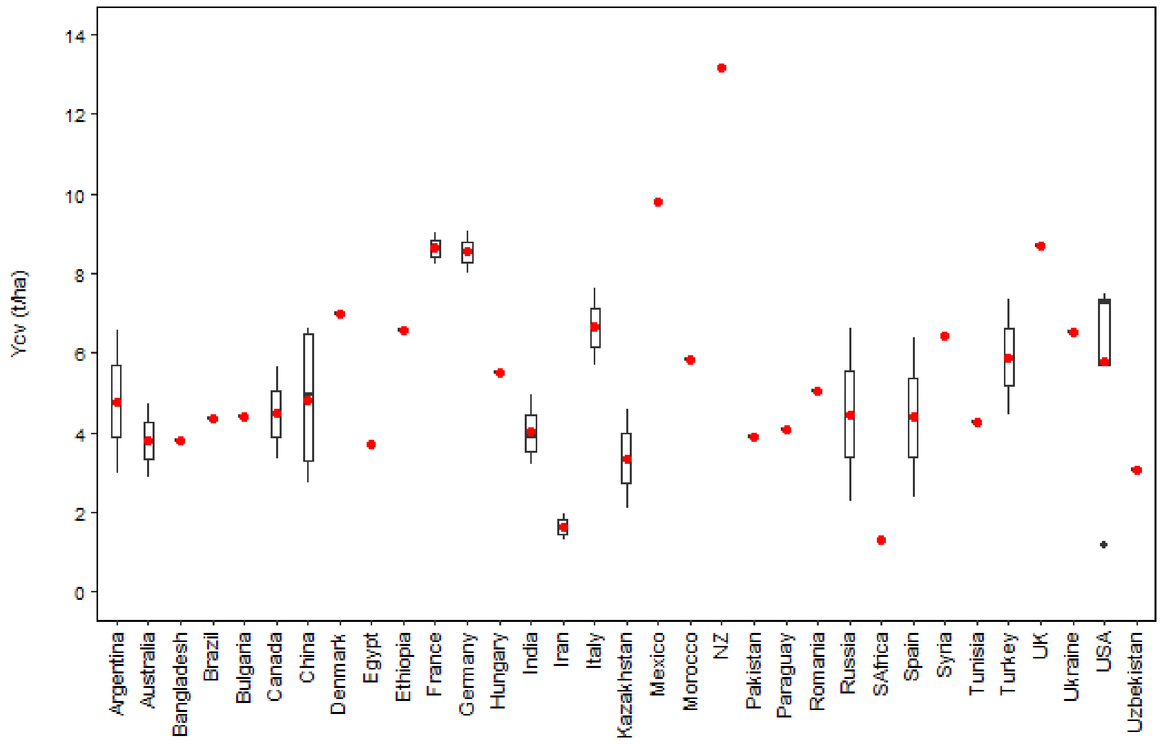

Fig. S7 Estimated potential yield of current wheat cultivars with optimal management (*Ycv*) in rainfed conditions under baseline climate (1985-2015) in different wheat producing countries. The boxplot is used for countries with several sites and represents the 5th percentile, 25th percentile, median, 75th percentile and 95th percentile over the study sites in a country, with the red circle showing the mean.

(a)

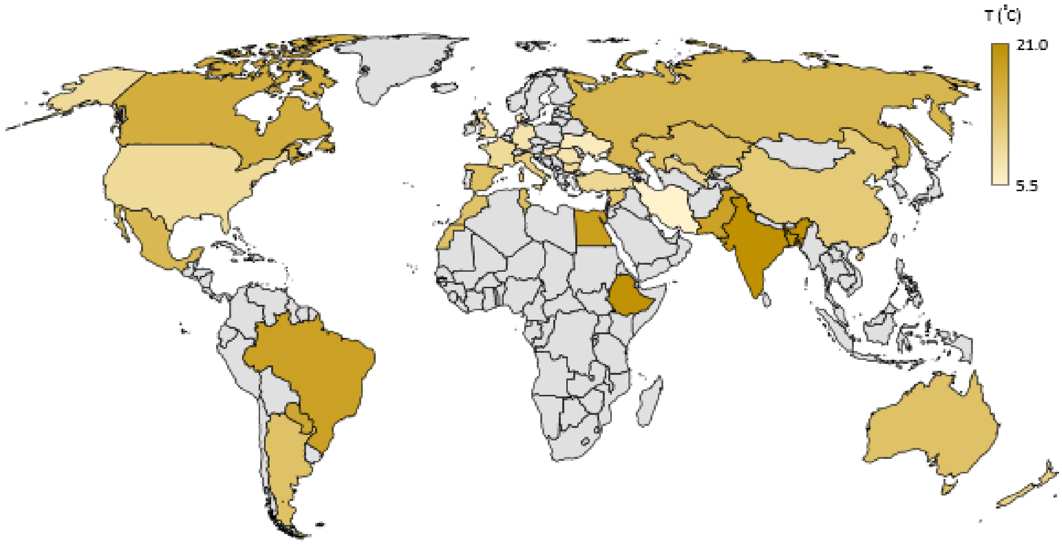

(b)

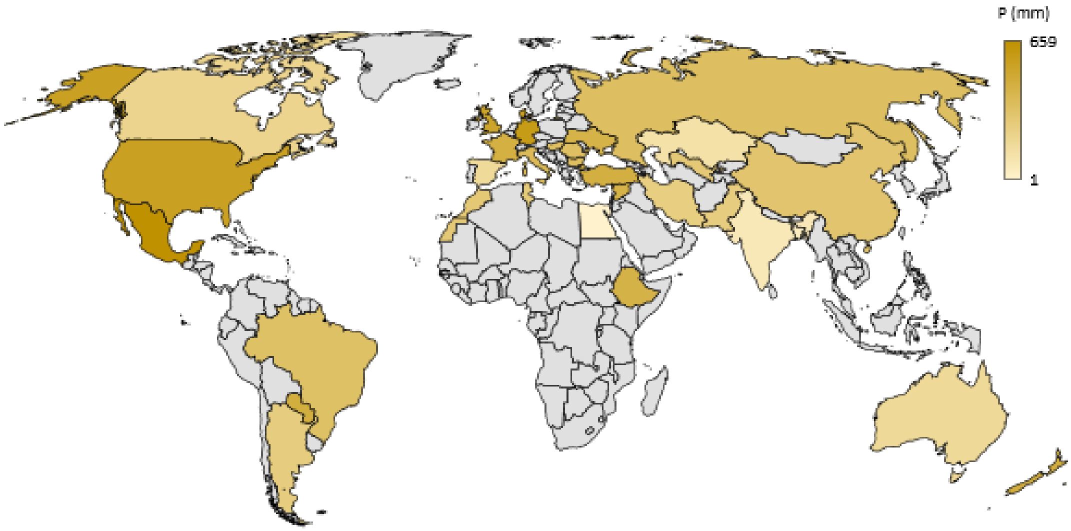

(c)

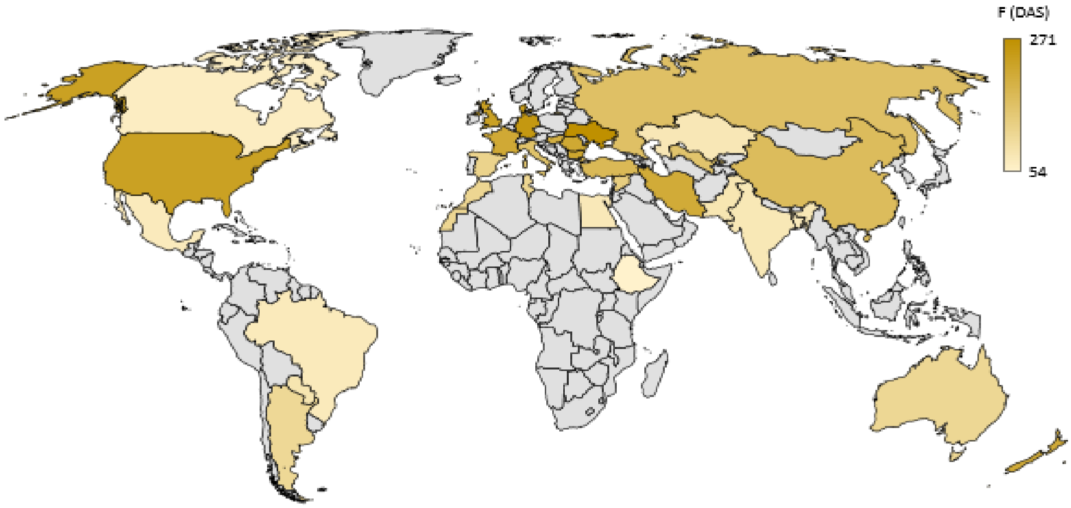

(d)

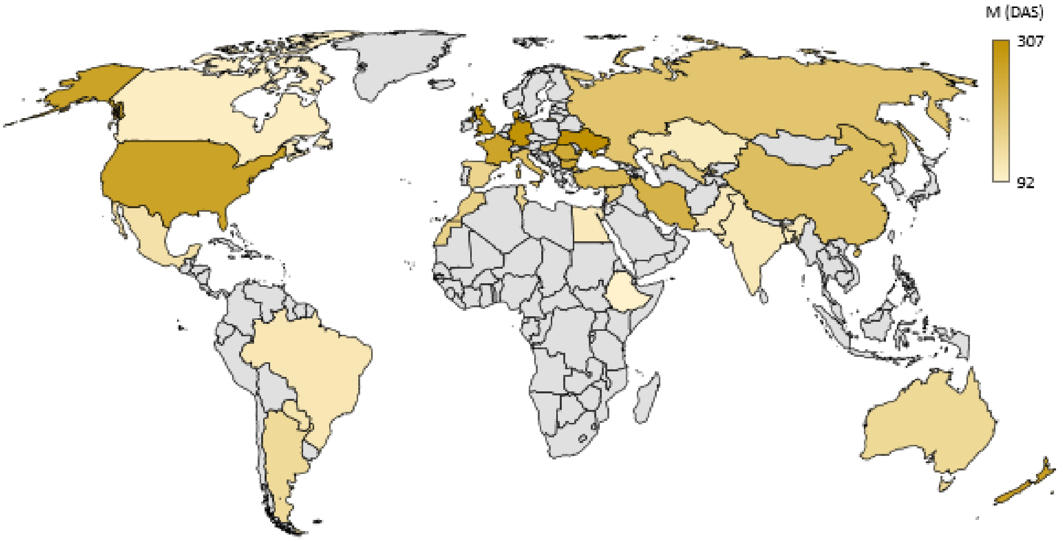

(e)

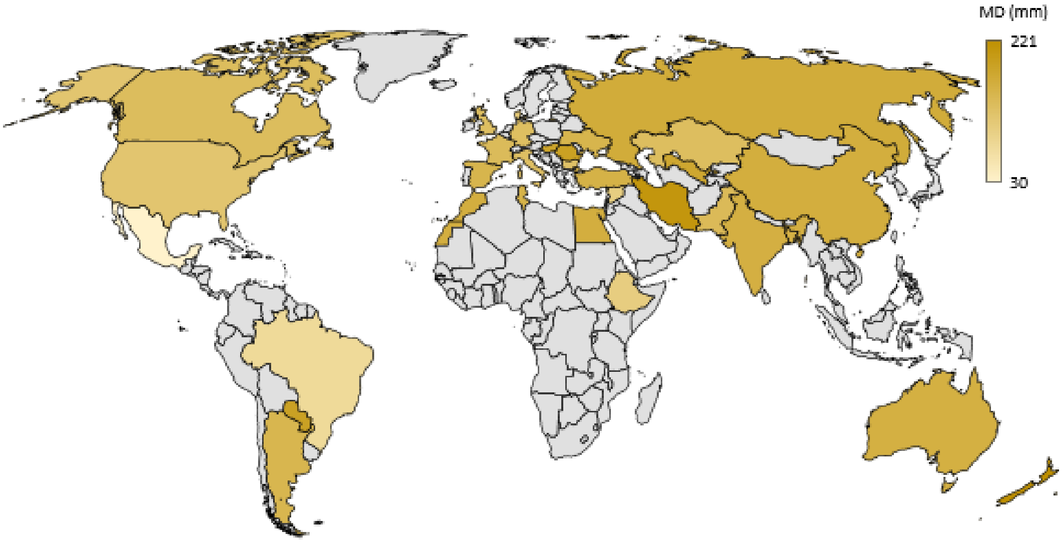

(f)

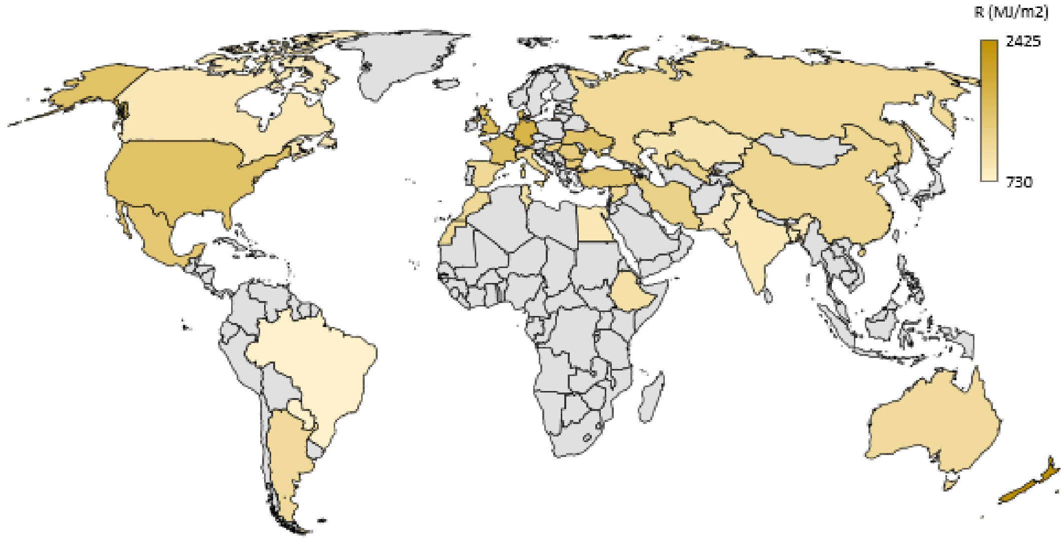

Fig. S8 Simulated growing season average air temperature (T, °C) (a), precipitation (P, mm) (b), flowering time (F, DAS: Days After Sowing) (c), maturity (M, DAS: Days After Sowing) (d), maximum soil water deficit (MD, mm) (e) and intercepted solar radiation (R, MJ/m^2) (f) for current wheat cultivars in major producing countries in rainfed condition with optimal management under baseline (1985-2015).

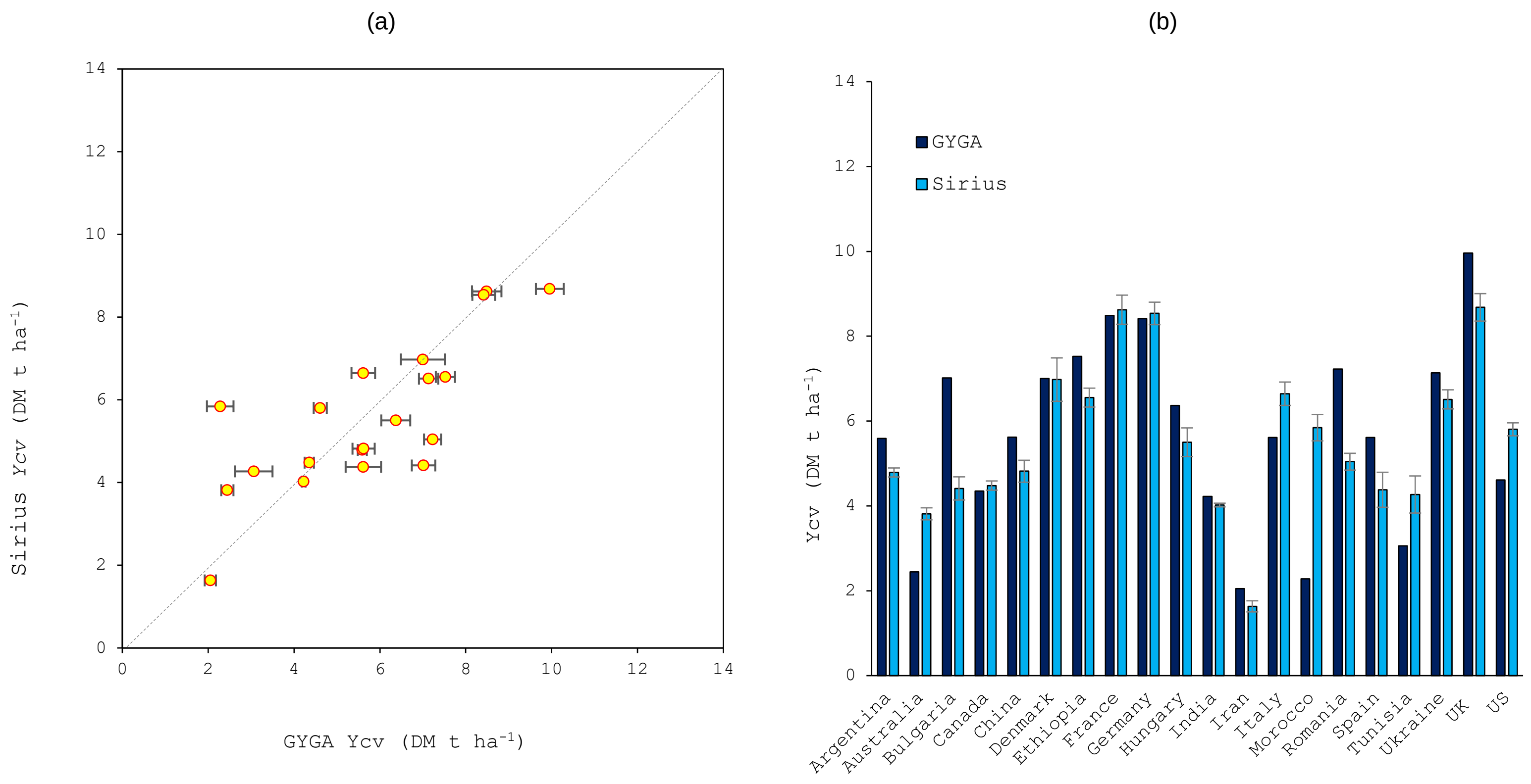

Fig. S9 Potential yield of current wheat cultivars with optimal management (*Ycv*) in rainfed condition under baseline climate estimated by Sirius, and similar estimates by GYGA (https://www.yieldgap.org/)(GYGA 2024). (a) 1:1 plot and (b) bar chart. Error bars are variation in yield estimates by GYGA (standard error) due to year variation for a country.

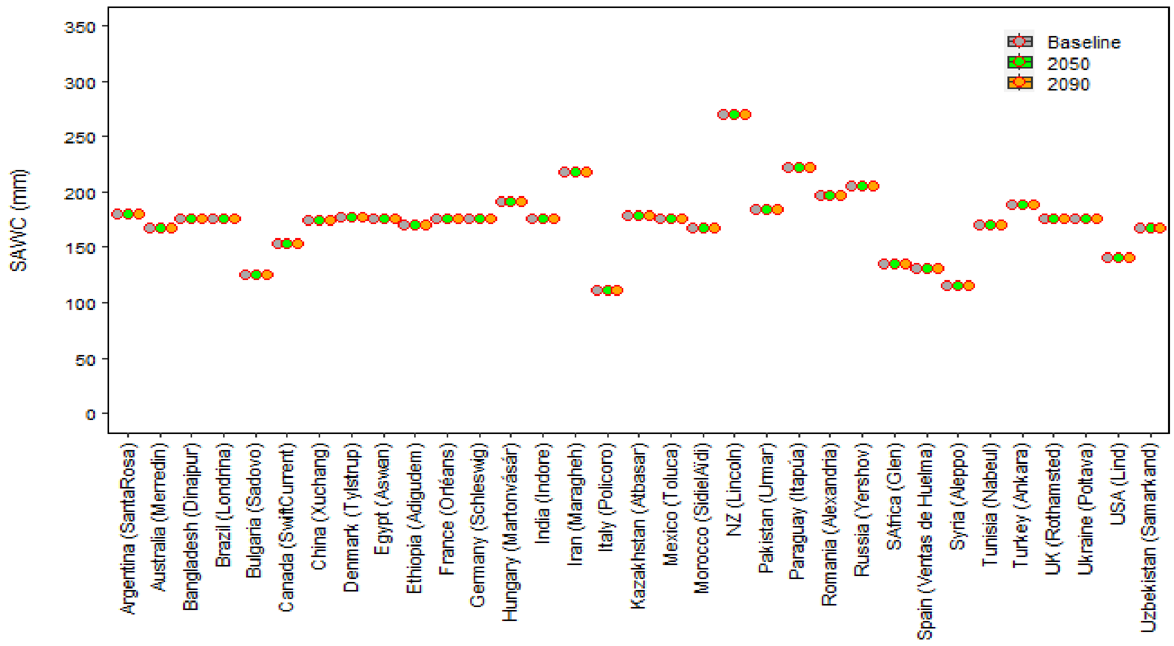
SAWC (mm)
Baseline
2050
2090
0
50
100
150
200
250
300
350
Argentina (SantaRosa)
Australia (Merredin)
Bangladesh (Dinajpur)
Brazil (Londrina)
Bulgaria (Sadovo)
Canada (SwiftCurrent)
China (Xuchang)
Denmark (Tylstrup)
Egypt (Aswan)
Ethiopia (Adigudem)
France (Orléans)
Germany (Schleswig)
Hungary (Martonvásár)
India (Indore)
Iran (Maragheh)
Italy (Policoro)
Kazakhstan (Atbasar)
Mexico (Toluca)
Morocco (SidielAidi)
NZ (Lincoln)
Pakistan (Urmar)
Paraguay (Itapúa)
Romania (Alexandria)
Russia (Yershov)
SAfrica (Glen)
Spain (Ventas de Huelma)
Syria (Aleppo)
Tunisia (Nabeul)
Turkey (Ankara)
UK (Rothamsted)
Ukraine (Poltava)
USA (Lind)
Uzbekistan (Samarkand)

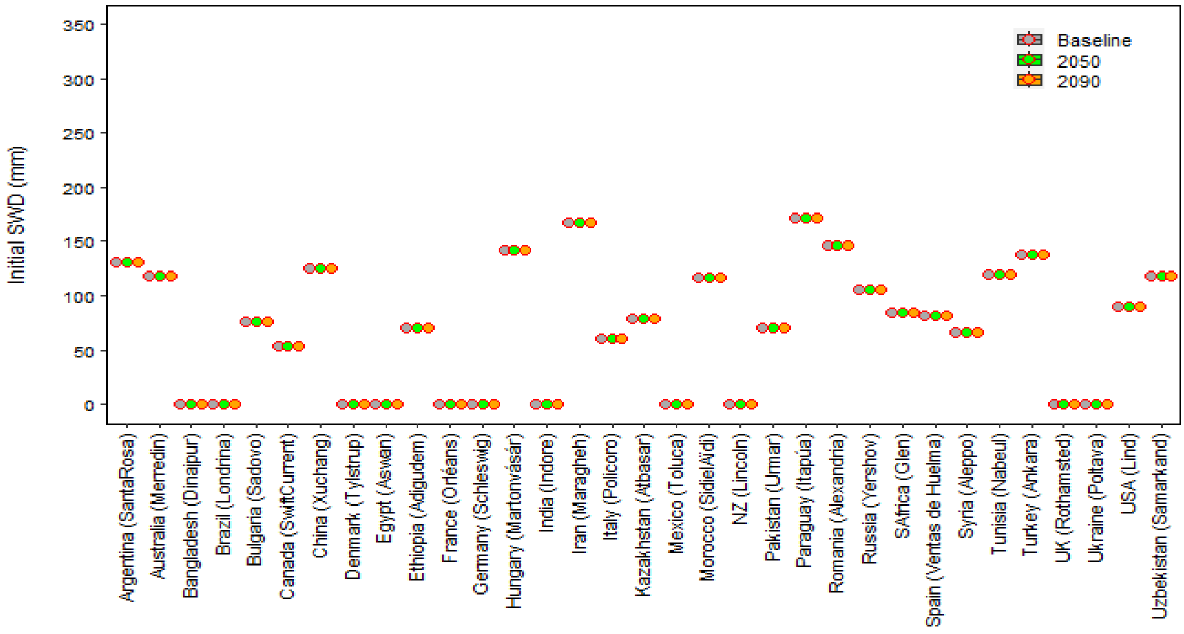
Initial SWD (mm)
Baseline
2050
2090
0
50
100
150
200
250
300
350
Argentina (SantaRosa)
Australia (Merredin)
Bangladesh (Dinajpur)
Brazil (Londrina)
Bulgaria (Sadovo)
Canada (SwiftCurrent)
China (Xuchang)
Denmark (Tylstrup)
Egypt (Aswan)
Ethiopia (Adigudem)
France (Orléans)
Germany (Schleswig)
Hungary (Martonvásár)
India (Indore)
Iran (Maragheh)
Italy (Policoro)
Kazakhstan (Atbasar)
Mexico (Toluca)
Morocco (SidielAidi)
NZ (Lincoln)
Pakistan (Urmar)
Paraguay (Itapúa)
Romania (Alexandria)
Russia (Yershov)
SAfrica (Glen)
Spain (Ventas de Huelma)
Syria (Aleppo)
Tunisia (Nabeul)
Turkey (Ankara)
UK (Rothamsted)
Ukraine (Poltava)
USA (Lind)
Uzbekistan (Samarkand)

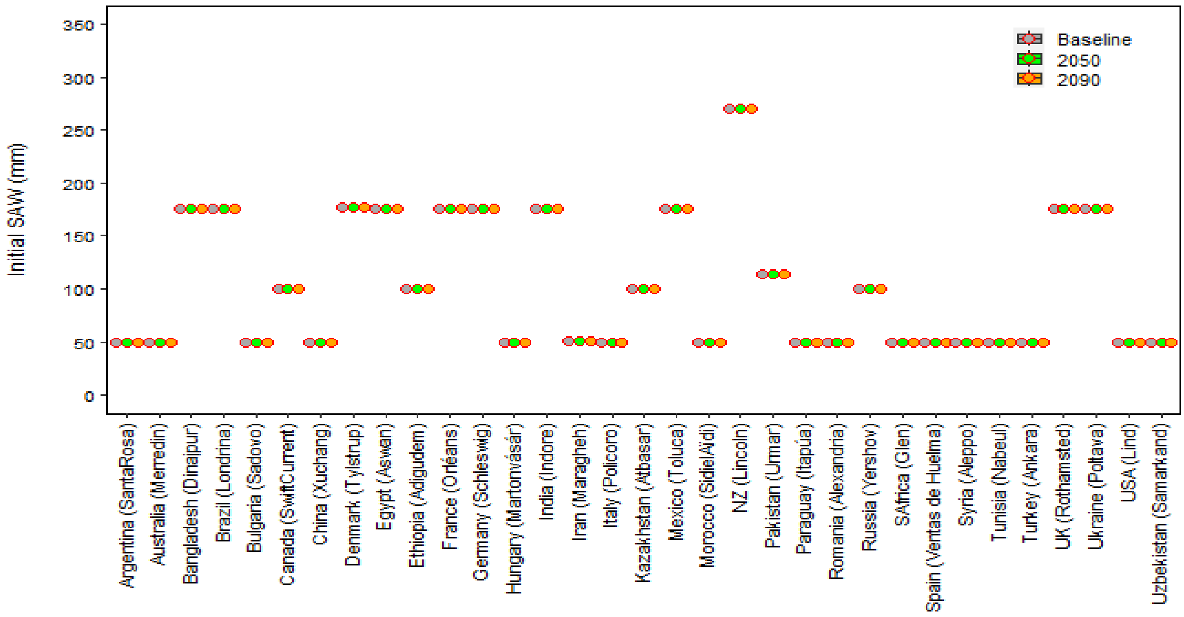
Initial SAW (mm)
Baseline
2050
2090
0
50
100
150
200
250
300
350
Argentina (SantaRosa)
Australia (Merredin)
Bangladesh (Dinajpur)
Brazil (Londrina)
Bulgaria (Sadovo)
Canada (SwiftCurrent)
China (Xuchang)
Denmark (Tylstrup)
Egypt (Aswan)
Ethiopia (Adigudem)
France (Orléans)
Germany (Schleswig)
Hungary (Martonvásár)
India (Indore)
Iran (Maragheh)
Italy (Policoro)
Kazakhstan (Atbasar)
Mexico (Toluca)
Morocco (SidielAidi)
NZ (Lincoln)
Pakistan (Urmar)
Paraguay (Itapúa)
Romania (Alexandria)
Russia (Yershov)
SAfrica (Glen)
Spain (Ventas de Huelma)
Syria (Aleppo)
Tunisia (Nabeul)
Turkey (Ankara)
UK (Rothamsted)
Ukraine (Poltava)
USA (Lind)
Uzbekistan (Samarkand)

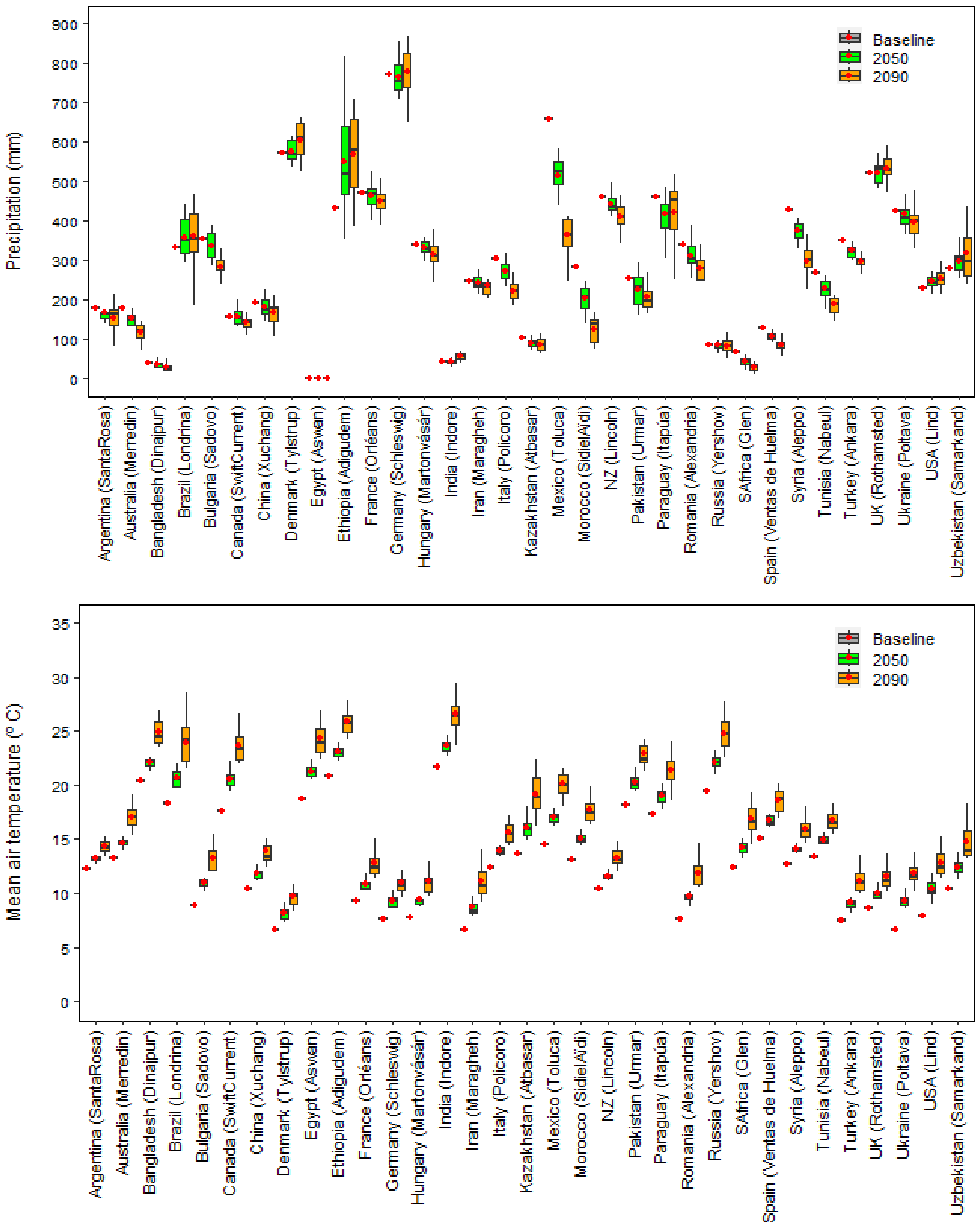

Fig. S10 Soil available water capacity (SAWC), initial soil water deficit (SWD), initial soil available water (SAW), growing season precipitation and air temperature, precipitation in major producing countries in rainfed condition with optimal management under baseline (1985-2015) and future climates (2050 and 2090) with atmospheric CO_2 concentrations of 363.8, 562.8 and 1001.8 ppm under baseline, 2050 and 2090 climates, respectively. Each box plot represents the 5th percentile, 25th percentile, median, 75th percentile and 95th percentile including mean (red circle) of simulations based on 15 global climate models from the CMIP6 ensemble at the extreme site in a country (see Methods).

References

Asseng S, Ewert F, Martre P et al (2015) Rising temperatures reduce global wheat production. Nat Clim Chang 5 (2):143-147. doi:https://doi.org/10.1038/nclimate2470

Asseng S, Ewert F, Rosenzweig C et al (2013) Uncertainty in simulating wheat yields under climate change. Nat Clim Chang 3 (9):827-832. doi:https://doi.org/10.1038/nclimate1916

Asseng S, Martre P, Maiorano A et al (2019) Climate change impact and adaptation for wheat protein. Glob Change Biol 25 (1):155-173. doi:https://doi.org/10.1111/gcb.14481

Brooks RJ, Semenov MA, Jamieson PD (2001) Simplifying Sirius: sensitivity analysis and development of a meta-model for wheat yield prediction. Eur J Agron 14 (1):43–60. doi:10.1016/s1161-0301(00)00089-7

Danabasoglu G (2019) NCAR CESM2 model output prepared for CMIP6 CMIP historical. Earth System Grid Federation. https://doi.org/10.22033/ESGF/CMIP6.7627

FAOSTAT (2021) Crop database. Food and Agriculture Organisation of the United Nations. http://www.fao.org/faostat/en/#data/QC.

Guarin JR, Martre P, Ewert F, Webber H et al (2022) Evidence for increasing global wheat yield potential. Environ Res Lett 17 (12):124045. doi:10.1088/1748-9326/aca77c

GYGA (2024) Global Yield Gap and Water Productivity Atlas. www.yieldgap.org (accessed on 11/7/2024).

Jamieson PD, Brooking IR, Semenov MA, MeMaster GS, White JW, Porter JR (2007) Reconciling alternative models of phenological development in winter wheat. Field Crop Res 103 (1):36–41. doi:10.1016/j.fcr.2007.04.009

Jamieson PD, Brooking IR, Semenov MA, Porter JR (1998a) Making sense of wheat development: a critique of methodology. Field Crop Res 55 (1):117–127. https://doi.org/10.1016/S0378-4290(97)00072-5

Jamieson PD, Semenov MA (2000) Modelling nitrogen uptake and redistribution in wheat. Field Crop Resh 68 (1):21–29. doi:10.1016/s0378-4290(00)00103-9

Jamieson PD, Semenov MA, Brooking IR, Francis GS (1998b) Sirius: a mechanistic model of wheat response to environmental variation. Eur J Agron 8 (3-4):161–179. https://doi.org/10.1016/S1161-0301(98)00020-3

Krasting JP, John JG, Blanton C et al (2018) NOAA-GFDL GFDL-ESM4 model output prepared for CMIP6 CMIP. Earth System Grid Federation. https://doi.org/10.22033/ESGF/CMIP6.1407

Lawless C, Semenov MA, Jamieson PD (2005) A wheat canopy model linking leaf area and phenology. Eur J Agron 22 (1):19-32. https://doi.org/10.1016/j.eja.2003.11.004

Lee W-L, Liang H-C (2020) AS-RCEC TaiESM1.0 model output prepared for CMIP6 CMIP 1pctCO2. Earth System Grid Federation. https://doi.org/10.22033/ESGF/CMIP6.9702

Liu B, Asseng S, Muller C et al (2016) Similar estimates of temperature impacts on global wheat yield by three independent methods. Nat Clim Chang 6:1130-1136. https://doi.org/10.1038/nclimate3115

Liu B, Martre P, Ewert F et al (2019) Global wheat production with 1.5 and 2.0°C above pre-industrial warming. Glob Change Biol 25 (4):1428-1444. https://doi.org/10.1111/gcb.14542

Martre P, Wallach D, Asseng S et al (2015) Multimodel ensembles of wheat growth: many models are better than one. Glob Change Biol 21 (2):911-925. https://doi.org/10.1111/gcb.12768

Nasa Goddard Institute for Space Studies (2018) NASA-GISS GISS-E2.1G model output prepared for CMIP6 ISMIP6. Earth System Grid Federation. https://doi.org/10.22033/ESGF/CMIP6.2066

Peano D, Lovato T, Materia S (2020) CMCC CMCC-ESM2 model output prepared for CMIP6 LS3MIP. Earth System Grid Federation. https://doi.org/10.22033/ESGF/CMIP6.13165

Roberts M (2017) MOHC HadGEM3-GC31-LL model output prepared for CMIP6 HighResMIP. Earth System Grid Federation. https://doi.org/10.22033/ESGF/CMIP6.1901

Semenov MA (2021) Sirius crop model (Sirius 2018). Zenodo. https://doi.org/10.5281/zenodo.4572624.

Semenov MA, Stratonovitch P, Alghabari F, Gooding MJ (2014) Adapting wheat in Europe for climate change. J Cereal Sci 59 (3):245-256. http://dx.doi.org/10.1016/j.jcs.2014.01.006

Senapati N, Halford NG, Semenov MA (2021) Vulnerability of European wheat to extreme heat and drought around flowering under future climate. Environ Res Lett 16, 024052. doi:10.1088/1748-9326/abdcf3

Senapati N, Semenov MA, Halford NG et al (2022) Global wheat production could benefit from closing the genetic yield gap. Nat Food 3 (7):532-541. doi:10.1038/s43016-022-00540-9

Senapati N, Stratonovitch P, Paul MJ, Semenov MA (2019) Drought tolerance during reproductive development is important for increasing wheat yield potential under climate change in Europe. J Exp Bot 70 (9):2549–2560. https://doi.org/10.1093/jxb/ery226

Shiogama H, Abe M, Tatebe H (2019) MIROC MIROC6 model output prepared for CMIP6 ScenarioMIP. Earth System Grid Federation. https://doi.org/10.22033/ESGF/CMIP6.898

Stratonovitch P, Semenov MA (2015) Heat tolerance around flowering in wheat identified as a key trait for increased yield potential in Europe under climate change. J Exp Bot 66 (12):3599-3609. doi:10.1093/jxb/erv070

Swart NC, Cole JNS, Kharin VV et al (2019) CCCma CanESM5 model output prepared for CMIP6 ScenarioMIP. Earth System Grid Federation. https://doi.org/10.22033/ESGF/CMIP6.1317

Tang Y, Rumbold S, Ellis R, Kelley D, Mulcahy J, Sellar A, Walton J, Jones C (2019) MOHC UKESM1.0-LL model output prepared for CMIP6 CMIP historical. Earth System Grid Federation. https://doi.org/10.22033/ESGF/CMIP6.6113

Voldoire A (2018) CNRM-CERFACS CNRM-CM6-1 model output prepared for CMIP6 CMIP. Earth System Grid Federation. https://doi.org/10.22033/ESGF/CMIP6.1375

Volodin E, Mortikov E, Gritsun A, Lykossov V, Galin V, Diansky N, Gusev A, Kostrykin S, Iakovlev N, Shestakova A, Emelina S (2019) INM INM-CM5-0 model output prepared for CMIP6 CMIP piControl. Earth System Grid Federation. https://doi.org/10.22033/ESGF/CMIP6.5081

Wang E, Martre P, Zhao Z et al (2017) The uncertainty of crop yield projections is reduced by improved temperature response functions. Nat Plant 3:17102. https://doi.org/10.1038/nplants.2017.102

Wieners K-H, Giorgetta M, Jungclaus J et al (2019) MPI-M MPI-ESM1.2-LR model output prepared for CMIP6 ScenarioMIP ssp245. Earth System Grid Federation. https://doi.org/10.22033/ESGF/CMIP6.6693

Wu T, Chu M, Dong M, Fang Y et al (2018) BCC BCC-CSM2MR model output prepared for CMIP6 CMIP piControl. Earth System Grid Federation. https://doi.org/10.22033/ESGF/CMIP6.3016

Yukimoto S, Koshiro T, Kawai H et al (2019) MRI MRI-ESM2.0 model output prepared for CMIP6 CMIP. Earth System Grid Federation. https://doi.org/10.22033/ESGF/CMIP6.621

Ziehn T, Chamberlain M, Lenton A et al (2019) CSIRO ACCESS-ESM1.5 model output prepared for CMIP6 CMIP. Earth System Grid Federation. https://doi.org/10.22033/ESGF/CMIP6.2288
